# Metabolic profiling of single cells by exploiting NADH and FAD fluorescence via flow cytometry

**DOI:** 10.1016/j.molmet.2024.101981

**Published:** 2024-07-04

**Authors:** Ariful Haque Abir, Leonie Weckwerth, Artur Wilhelm, Jana Thomas, Clara M. Reichardt, Luis Munoz, Simon Völkl, Uwe Appelt, Markus Mroz, Raluca Niesner, Anja Hauser, Rebecca Sophie Fischer, Katharina Pracht, Hans-Martin Jäck, Georg Schett, Gerhard Krönke, Dirk Mielenz

**Affiliations:** 1 Division of Molecular Immunology, Department of Internal Medicine 3, Friedrich-Alexander-Universität Erlangen-Nürnberg and Universitätsklinikum Erlangen, Nikolaus-Fiebiger-Center, Glückstr. 6, 91054 Erlangen, Germany; 2 Department of Internal Medicine 3 - Rheumatology and Immunology, Friedrich-Alexander-University Erlangen-Nürnberg (FAU) and Universitätsklinikum Erlangen, Erlangen, Germany; 3 Deutsches Zentrum für Immuntherapie (DZI), Friedrich-Alexander-University Erlangen-Nürnberg and Universitätsklinikum Erlangen, Erlangen, Germany; 4 Department of Internal Medicine 5, Hematology and Oncology, Friedrich-Alexander-Universität Erlangen-Nürnberg and Universitätsklinikum Erlangen, Ulmenweg 18, 91054 Erlangen, Germany; 5 Flow cytometry core unit, Friedrich-Alexander-Universität Erlangen-Nürnberg, Glückstr. 6, 91054 Erlangen, Germany; 6 Deutsches Rheumaforschungszentrum Berlin, Biophysikalische Analytik, Charitéplatz 1, 10117 Berlin, Germany; 7 Freie Universität Berlin, Dynamisches und funktionelles in vivo Imaging, Adresse: Oertzenweg 19b, 14163 Berlin, Germany; 8 Medizinische Klinik mit Schwerpunkt Rheumatologie und Klinische Immunologie, Charité - Universitätsmedizin Berlin, Charitéplatz 1, 10117 Berlin, Germany; 9 Deutsches Rheumaforschungszentrum Berlin, Immundynamik, Charitéplatz 1, 10117 Berlin, Germany

**Keywords:** NADH, FAD, Metabolism, Fluorescence, Real-time, Flow cytometry

## Abstract

**Objective:**

The metabolism of different cells within the same microenvironment can differ and dictate physiological or pathological adaptions. Current single-cell analysis methods of metabolism are not label-free.

**Methods:**

The study introduces a label-free, live-cell analysis method assessing endogenous fluorescence of NAD(P)H and FAD in surface-stained cells by flow cytometry.

**Results:**

OxPhos inhibition, mitochondrial uncoupling, glucose exposure, genetic inactivation of glucose uptake and mitochondrial respiration alter the optical redox ratios of FAD and NAD(P)H as measured by flow cytometry. Those alterations correlate strongly with measurements obtained by extracellular flux analysis. Consequently, metabolically distinct live B-cell populations can be resolved, showing that human memory B-cells from peripheral blood exhibit a higher glycolytic flexibility than naïve B cells. Moreover, the comparison of blood-derived B- and T-lymphocytes from healthy donors and rheumatoid arthritis patients unleashes rheumatoid arthritis-associated metabolic traits in human naïve and memory B-lymphocytes.

**Conclusions:**

Taken together, these data show that the optical redox ratio can depict metabolic differences in distinct cell populations by flow cytometry.

## Highlights

• Real-time simultaneous metabolic analysis of different cell populations.
• Label-free detection of the optical redox ratio of FAD/NADH fluorescence via flow cytometry.
• Correlation with Seahorse extracellular flux analysis and enzymatic NAD(P)H detection.
• Applicable to human T cell lymphoma, primary mouse and human lymphocytes.
• Distinction of naïve and memory B cell populations in healthy and inflamed backgound.

## Abbreviations

Acetyl-CoA: Acetyl Coenzyme A
ACPA: antibodies directed against citrullinated peptides
ATP: adenosine triphosphate
CLL: chronic lymphocytic leukemia
2-DG: 2-Deoxyglucose
DLBCL: diffuse large B cell lymphoma
DNT: dominant negative Twinkle
ECAR: extracellular acidification rate
ETC: electron transport chain
FAD: Flavin adenine dinucleotide
FCCP: carbonyl cyanide m-chlorophenyl hydrazine
FI: fluorescence intensity
FL: fluorescence
FLIM: fluorescence lifetime imaging
FSc: Forward Scatter
GFP: green fluorescent protein
HD: healthy donor
IMM: inner mitochondrial membrane
Mn: mean
Md: median
mtDNA: mitochondrial DNA
NADH: Nicotinamide adenine dinucleotide
OxPhos: oxidative phosphorylation
GST: glyco stress test
MST: mito stress test
ORR: optical redox ratio
OxPhos: oxidative phosphorylation
RA: rheumatoid arthritis
ROS: reactive oxygen species
OCR: oxygen consumption rate
SSc: Side scatter
SDH: succinate dehydrogenase

## 1 Introduction

Autoimmunity, cancer, obesity and diabetes are associated with abnormal cellular bioenergetics in various cell types (reviewed in [1]). The most fundamental parameters of cellular bioenergetics are respiration and anaerobic-like glycolysis, hereafter termed glycolysis. Cellular metabolism controls cell fate decisions and immune cell effector functions and vice versa, cell-intrinsic programs control metabolism [2,3]. In addition, changing environments induce the rewiring of redox networks [4]. For instance, germinal center responses, plasma cell differentiation and humoral immunity require oxidative phosphorylation (OxPhos) in B cells [5,6], while pre germinal center B cell proliferation depends on pyruvate reduction by lactate dehydrogenase A [7]. In pathologic conditions, such as rheumatoid arthritis (RA), T cells with a short-lived, pro-inflammatory effector phenotype appear in the blood. This correlates with mitochondrial DNA (mtDNA) damage [8]. Hence, identifying immune cell metabolic phenotypes discriminating healthy from pathological conditions may be a key tool for diagnostic purposes. Indeed, alterations in cell metabolism have been linked to disease for a long: Otto Warburg has found that tumor cells produce lactate even under aerobic conditions [8], a phenomenon that also may take place in rapidly proliferating non-transformed cells or in cells with defects of complex I assembly [9].

To better depict changes in cellular metabolism in disease, simple and accurate methods capable of measuring complex and limited samples - preferably in high throughput and non-invasive manner - are warranted. Measuring O_2_ partial pressure (pO_2_) to determine the degree of oxygen consumption in cell culture can quantify mitochondrial respiration. Glycolysis is the oxygen-independent conversion of glucose to pyruvate and further, lactate. Cells secrete lactate, thereby, acidifying the surrounding medium. Hence, respiration and glycolysis can be measured via extracellular flux analyses (“Seahorse”) and are represented by oxygen consumption rate (OCR) and extracellular acidification rate (ECAR) [10]. This approach, while in principle being established since long [11], contributed to our understanding of how metabolic states impact immune cell function. However, significant numbers of cells are still required, which means that scaled-down methods need to be developed in order to assess low abundant cell populations.

A potential way to measure cell metabolism at single cell level is based on the measurement of Nicotinamide adenine dinucleotide (NADH) and Flavin adenine dinucleotide (FAD). Glycolysis yields two moles of adenosine triphosphate (ATP) per mol of glucose. The action of glyceraldehyde phosphate dehydrogenase in the glycolysis pathway generates in addition two moles of reduced NADH per mol of glucose [12]. Whereas NADH derived from glycolysis is required for the reduction of pyruvate to lactate, it is also an important co-factor for lipid and glutathione synthesis, thereby, contributing to cellular redox homeostasis [4]. Based on the cell type and activation state, pyruvate enters mitochondria. In the mitochondrial matrix, first pyruvate becomes oxidized via the generation of Acetyl Coenzyme A (Acetyl-CoA). Acetyl-CoA is further processed in multiple energy-favored reactions involving carboxylic acids, such as ketoglutarate, succinate or fumarate. These reactions also generate NADH, and finally, oxaloacetate [13]. The Krebs cycle fuels oxidative phosphorylation in mitochondria via succinate dehydrogenase (SDH), which oxidizes succinate to fumarate while reducing FAD to FADH_2_. Simultaneously, SDH catalyzes the reaction of ubichinone to ubichinol by re-oxidizing FADH_2_ to FAD [14,15]. Thereby, two protons and two electrons are transported across the inner mitochondrial membrane (IMM). Complex I oxidizes NADH and the electrons move along the ETC (ETC) complexes. During this process, and during lactate synthesis as well, NADH is reduced to NAD^+^. Complexes I-III pump the protons derived from NADH across the IMM and complex IV transfers electrons to O_2_, the essential terminal electron acceptor. Finally, Complex V utilizes the chemiosmotic gradient to produce ATP [12].

Consequently, the redox state of cells, defined as the relative amounts of reduced to oxidized NADH and FAD, respectively, can be determined as a surrogate for the cell's metabolic status. Enzymatic measurements can quantify NAD(P)H in cell lysates. However, the reduced form of NAD, NAD(P)H, and the oxidized form of FAD, are fluorescent upon excitation at 335 nm (reviewed in [1,16]). The fluorescence of NAD^+^ and FADH_2_ is much lower and typically not detectable [[17], [18], [19]]. Thus, the ratio of NAD^+^/[NADH^+^+NAD^+^] correlates (i) with mitochondrial activity and (ii) with the optical redox ratio (ORR) of FAD/[NAD(P)H + FAD] in multiple cell types and tissues [20,21]. Hence, the ORR represents a non-invasive indicator of cellular metabolic states directly at the single cell level and even at the subcellular level [1,18,22], while extracellular flux analysis reflects changes in the extracellular milieu of multiple cultured cells. Recently, the label-free measurement of NAD(P)H and FAD discriminating immune cell populations and glioma cells by flow cytometry has been established [23,24]. It was found that immune cells activated with specific stimuli increase NAD(P)H and that different purified immune cells exhibit different redox ratios [23]. The cellular responses to acute sequential changes, such as inhibition of OxPhos, mitochondrial uncoupling or acute glucose exposure that are routine applications in extracellular flux analysis have not been analyzed yet. However, these data would be needed in order to match the data with methods widely used in immunology such as metabolic flux and SCENITH [25]. Here, we combine the advantages of live cell metabolic measurements and flow cytometry. We quantify acute dynamic metabolic changes of the ORR by flow cytometry and correlate it with results obtained by extracellular flux analysis. Determining the ORR in complex samples, such as peripheral blood mononuclear cells, stained with multiple surface markers, uncovers previous unknown metabolic signatures in human memory B cells. Thus, this method allows the correlation of metabolic parameters with arbitrary phenotypes, even of multiple rare cell types within a single sample.

## 2 Materials & methods

### 2.1 Mice – breeding and housing

The mice for this study were housed under pathogen-free conditions in the Franz-Penzoldt-Zentrum or the Nikolaus-Fiebiger Center animal facility of the University of Erlangen-Nürnberg, Erlangen, Germany. All experimental procedures and ethical considerations were conducted in accordance with approved animal protocols by the government of Lower Franconia, Bavaria, Germany. The experiments involved both female and male mice at 8–14 weeks of age, and they were kept on a 12-hour light/dark cycle with free access to food and water, following governmental regulations.

To form the experimental group of B cell specific Glut1^KO^ mice, transgenic CD23Cre-mice were crossed with loxP-flanked Slc2a1-mice [26]. For the DNT mice, R26-K320E-TWINKLE loxP+/−mice [27] were crossed with CD23 CRE mice ([28]; kindly provided by Meinrad Busslinger). The CRE control mice were DNT^−/−^ CRE^+/−^ or Glut1^−/−^CRE^+/−^. WT (wild-type) animals utilized in the study were DNT^−/−^ or Glut1^−/−^ CRE^−/−^ littermates. Age and sex-matched mice of both sexes were used for the analyses and all of them were on the C57Bl/6 background.

### 2.2 Preparation of primary murine cells

Mice were sacrificed using CO_2_ and the spleen was transferred into cold 2% fetal calf serum (FCS) (in PBS). The minced spleen tissue was carefully homogenized through a 70 μM cell strainer (Falcon, Corning Life Sciences, #352350) using the end of a 2 ml syringe stamp. The resulting cell suspensions were transferred into a 15 ml falcon tube and pelleted by centrifuging at 470 g and 4 °C for 7 min. Erythrocytes were lysed in 5 ml Red blood cell lysis buffer (BioLegend; #420301) at room temperature for 5 min. The reaction was stopped with 5 ml of cold PBS-2% FCS buffer. The cell suspensions were filtered through a 30 μm CellTrics filter (Sysmex, #04-0042-2316) before centrifugation at 470 *g* and 4 °C for 7 min. The resulting cell pellets were resuspended in PBS-2% FCS buffer and the cell concentration was determined using the cell and viability assay (Solution 13) with the NucleoCounter NC-3000 (ChemoMetec) following the manufacturer's protocol.

### 2.3 Isolation of primary murine cells from spleen

Magnetic isolation of the B cells from splenic single-cell suspensions was performed under sterile conditions using the EasySep™ Mouse B Cell Isolation Kit (StemCell, #19854) following the manufacturer's specifications. The cell concentrations were determined using the cell and viability assay (Solution 13) with the NucleoCounter NC-3000 (ChemoMetec) according to the manufacturer's protocol. The quality of the B cell purification was assessed by flow cytometry using CD19 and B220 antibodies for surface staining. B cell enrichments after isolation were consistently above 95%.

### 2.4 In vitro cultivation of primary murine B cells

Isolated splenic B cells were cultured in complete R10 medium (RPMI1640, 10 % fetal calf serum (FCS), 2 mM glutamate, 1 mM sodium pyruvate, 50 U/ml penicillin G, 50 μg/ml streptomycin, 50 μM β-mercaptoethanol) at 37 °C and 5% CO2 for 72 h with the addition of 10 μg/ml lipopolysaccharide (LPS). The initial cell concentration was adjusted to 0.5 × 10^6^ cells/ml for each experiment.

### 2.5 Extracellular flux analysis (Seahorse)

Seahorse experiments were performed as described previously [6]. Briefly, the day prior to the experiments, cell plates were coated with 10 μg/ml Poly-l-Lysine in 1x Tris–EDTA buffer. Splenic naïve B cells were isolated and activated with LPS in vitro. After three days LPS blasts were seeded at a density of 2.5 × 10^5^ cells/well and measured at least in triplicates. Extracellular flux analysis was performed as described [6]. Mito Stress Test, Glyco Stress Test and calculations were performed following the guidelines provided by the manufacturer (Agilent) and using WAVE software.

### 2.6 Flow cytometry

For the flow cytometric analysis, the cell number was adjusted to 0.5–2 × 10^6^ cells per FACS tube in a volume of 500 μl per sample. For the murine cells, before labeling surface proteins, non-specific antibody binding was blocked by using a 1:100 dilution of unlabeled anti-CD16/CD32 (Invitrogen, Cat. No.: 14-0161-86) in FACS buffer (consisting of PBS with 2% FCS) for 15 min at 4 °C. Cells were washed with FACS buffer and stained for 15 min at 4 °C with the appropriate amount of the following fluorophore-conjugated antibodies: TACI/CD267 APC (1:400), CD138 PECy7 (1:1500), GL-7 FITC (1:100), B220/CD45R PerCP Cy5.5 (1:200), CD19 APCFire750 (1:200).

For human cells, about 2 × 10^6^ – 4 × 10^6^ cells were harvested for the flow cytometric analysis. Then the cells were washed with FACS buffer containing PBS, 2% FCS, and 1 mM EDTA. Afterwards, human BD Fc Block™ (BD, Cat. No.: 564219, Dilution: 1:100) was introduced to the samples and was incubated for 10 min. After that cells were incubated with the following antibodies for 30 min: CD4 APC - mouse anti-human (BioLegend, Cat. No.: 317416, Dilution: 5:100), CD20 APC Cy7 - mouse anti-human (BioLegend, Cat. No.: 302314, Dilution: 1:20), CD38 PE Cy7 - mouse anti-human (BioLegend, Cat. No.: 356608, Dilution: 1:20), IgD rPE - goat anti-human (SouthernBiotech, Cat. No.: 2032-09, Dilution: 1:25), CD27 FITC - mouse anti-human (Dako, Cat. No.: F7178, Dilution: 1:10). After 30 min, cells were washed once with FACS buffer and one more time with the Seahorse XF RPMI medium pH7.4 (Agilent; Cat. No.: 103576-100) and/or Seahorse Mito Stress Test or Glucose Stress Test medium unless mentioned otherwise. Finally, cells were incubated at 37 °C and 5% CO_2_ in the corresponding medium for 45 min–1 h before the flow cytometric analysis. The experiments were consequently performed at constant room temperature (20–22 °C) in a climatized room with the same flow cytometer, with cells, media and inhibitors equilibrated to this temperature. Cells were analyzed with a BD LSR Fortezza machine utilizing high-speed flow settings. The cell concentration was carefully maintained to ensure each reading had a runtime of 10–15 s, resulting in a negligible reduction in sample volume. The fluorescence of NADH and FAD was measured by exciting the cells with a 355 nm UV laser and the emission was detected with bandpass filters for BP 450/50 (NADH) and BP 560/40 (FAD). The other fluorescent dyes were chosen so as not to have any interference with the measurement of NADH and FAD fluorescence. Flow cytometry data was analyzed with the Beckman Coulter Kaluza Analysis 2.1. Palmitate was added to the cells as BSA-Palmitate complex (https://wiki.oroboros.at/index.php/Palmitate). Table 1 depicts the stock and working concentrations used for metabolic assays (see Table 2).

**Table 1:** Stock and working concentrations of inhibitors and nutrients This table presents the chemicals and nutrients used for flow cytometry in this study. Cocnetrations of stocks, working concentrations and volumes are indicated.

Name	Stock concentration	Final concentration	Volume added to the sample	Sample volume
Glucose	500 mM (Seahorse XF basal medium)	10 mM	10 μl	490 μl
Glutamine	100 mM	2 mM	10 μl	490 μl
Oligomycin A	500 μM (Seahorse XF basal medium, diluted from a 1 mM Stock in DMSO)	10 μM	10 μl	490 μl
FCCP	250 μM (Seahorse XF basal medium, diluted from a 1 mM Stock in DMSO)	5 μM	10 μl	490 μl
Rotenone	50 μM (Seahorse XF basal medium, diluted from a 3.76 mM Stock in DMSO)	1 μM	10 μl	490 μl
Antimycin A	50 μM (Seahorse XF basal medium, diluted from a 1.267 mM Stock in DMSO)	1 μM	10 μl	490 μl
2DG	2500 mM (Seahorse XF basal medium)	100 mM	20 μl	480 μl
Palmitate + BSA	10 mM (Seahorse XF basal medium)	200 μM	10 μl	490 μl
FK 866	100 μM (Seahorse XF basal medium, diluted from a diluted from a 1 mM Stock in DMSO)	1 μM	5 μl	495 μl

**Table 2:** Description, source and identifier antibodies and chemicals. This table presents the chemicals, nutrients, materials and software used in this study.

Reagent or resource	Source	Identifier
**Antibodies Mouse**		
Anti TACI/CD267 (APC, clone eBio8F10-3)	eBioscience	Cat# 17-5942-82, RRID:AB_842758
Anti CD19 (APCFire750, clone 6D5)	Biolegend	Cat# 115558, RRID:AB_2572120
Anti B220/CD45R (PerCP Cy5.5, clone Ra3-6 b)	eBioscience	Cat# 45-0452-80 RRID:AB_906234
Anti CD138 (PECy7, clone 281-2)	Biolegend	Cat# 142513, RRID:AB_2562197
Anti GL-7 (FITC, clone GL-7)	BD Biosciences	Cat# 553666 AB_394981
**Antibodies Human**		
Anti IgD rPE	SouthernBiotech	Cat# 2032-09
Anti CD20 APC-Cy7	BioLegend	Cat# 302314
Human BD Fc Block™	BD Bioscience	Cat# 564219
Anti CD4 APC	BioLegend	Cat # 317416
CD27 FITC	Dako	Cat# F7178
CD38 PE Cy7	BioLegend	Cat# 356608
**Experimental Models: Organisms/Strains**		
R26-K320E-TWINKLE loxP+/−	Baris et al., 2015	N/A
Tg(Fcer2a-cre)5Mbu	Kwon et al., 2008	N/A
SLC2A1 loxP+/−	Young et al., 2011	N/A
**Chemicals, Peptides, and Recombinant Proteins**		
RPMI1640	Gibco - Thermo Fisher Scientific	Cat# 31870-25
l-glutamine	Gibco - Thermo Fisher Scientific	Cat# 25030-24
Penicillin/Streptomycin	Gibco - Thermo Fisher Scientific	Cat# 15140-122
β-mercaptoethanol	Gibco - Thermo Fisher Scientific	Cat# 31350-010
Sodium Pyruvate	Gibco - Thermo Fisher Scientific	Cat# 11360-039
Glucose	Carl Roth	Cat# HN06.3
Poly-l-Lysine	Sigma	Cat# P8920
FK866	Sigma	Cat# 481908
Sodium Palmitate	Sigma	Cat# P9767
Bovine serum albumin	Carl Roth	Cat# 8076.4
Oligomycin A	Sigma	Cat# 75351
Carbonyl cyanide-p-trifluoromethoxy-phenylhydrazone FCCP	Sigma	Cat# C2920
Lipopolysaccharide	Sigma	Cat# L3024
Rotenone	Sigma	Cat# R8875
Antimycin A	Sigma	Cat# A-8674
2-DG	Sigma	Cat# D8375
XF RPMI Assay medium [pH 7,4]	Agilent	Cat# 103576-100
XF Calibrant buffer	Agilent	Cat# 100840-000
Value FBS	Gibco	
CellCount and Viability Solution 13	Chemometec	Cat# 910-3013
EasySep™ Mouse B Cell Isolation Kit	StemCell	Cat# 19854
**Software and Algorithms**		
GraphPad Prism 9	Graphpad	https://www.graphpad.com/scientific-software/prism/
Seahorse Wave Desktop Software	Agilent	https://www.agilent.com/en/product/cell-analysis/real-time-cell-metabolic-analysis/xf-software/seahorse-wave-desktop-software-740897
Kaluza	Beckman Coulter	https://www.beckman.de/flow-cytometry/software/kaluza

### 2.7 Bioluminescent measurement of NAD^+^ and NADH

The cells were subjected to treatment for 10 min, followed by the transfer of 50 μl of each sample to a white-walled 96-well plate (Thermo Fisher, # 136101). Subsequently, immediate lysis was achieved by adding 50 μl of 1% DTAB (Merck Millipore, #D5047) in 0.2 M NaOH (Merck Millipore, #106462). The resulting solutions were divided equally into two wells each. To deplete NADH, 25 μl of 0.4 M HCl (Merck Millipore, #113386) was added to one well, while the other well remained untreated to deplete NAD^+^. The plate was then incubated at 60 °C for 15 min. After incubation, the plate was allowed to equilibrate to room temperature for 10 min. Subsequently, 25 μl of 0.5 M Trizma base (Merck Millipore, #T1503-25G) was added to the NADH-depleted samples, while 50 μl of HCl/Trizma solution (0.4 M HCl & 0.5 M Trizma Base, 1:2) was added to the NAD^+^-depleted samples. Next, 50 μl from each well was transferred to another plate, and 50 μl of NAD/NADH-Glo™ Detection Reagent (Promega, #G9071) was added to each well. The plate was incubated at room temperature for 45 min. Subsequently, luminescence was measured using the GloMax® Discover Microplate Reader.

### 2.8 Cell lines

Jurkat (Clone E6-1) cells were purchased from ATCC (TIB-152™), thawed and cultured in Gibco™ RPMI 1640 medium (Thermo Fisher Scientific, Cat. No: 11875093) supplemented with 10% heat-inactivated fetal bovine serum (Gibco™, Thermo Fisher Scientific), 2 mM l-Glutamine (Thermo Fisher Scientific, Cat. No: 25030024), 50 U/ml penicillin G, 50 μg/ml streptomycin, 50 μM β-mercaptoethanol. Cells were cultured in the incubator (at 37 °C, and 5% CO_2_) at a density of 10^6^ cells/ml in a CELLSTAR® TC T75 flask (Greiner, Cat. No.: 658175) until they were prepared for the corresponding experiments.

### 2.9 Peripheral blood mononuclear cell isolation

Venous blood from 12 healthy subjects was collected upon approval by the Ethics Committee of the University Clinic Erlangen. The 12 healthy subjects were randomly distributed between the experiments. The peripheral blood mononuclear cells (PBMC) were freshly isolated by standard density gradient centrifugation. Briefly, the blood samples were diluted 1:1 with PBS. Then they were gently pipetted into a 50 ml tube containing 15 ml of Pancoll human (density: 1.077 g/ml, PAN-Biotech, Cat. No.: P04-60125) and centrifuged. After centrifugation, cells were cryopreserved in liquid nitrogen. The frozen samples were thawed in a cell culture medium (RPMI1640, 10 % fetal calf serum (FCS), 2 mM glutamate, 1 mM sodium pyruvate, 50 U/ml penicillin G, 50 μg/ml streptomycin, 50 μM β-mercaptoethanol) and were kept in the incubator (at 37 °C and 5% CO_2_) overnight before preparing for the subsequent experiment.

RA patients were undergoing regular outpatient treatment at the Department of Medicine 3 of the University Clinic Erlangen. The inclusion of suitable patients for this cohort was based on previously generated medical reports and existing patient data, meeting the 2010 ACR/EULAR diagnostic criteria of RA (Supplemental Table 1). Furthermore, RA patients had to be seropositive based on the presence of a positive test for anti-citrullinated peptide antibodies (ACPA). Blood sample collection took place under informed consent and approval by the ethics committee of the University Hospital Erlangen during scheduled monitoring appointments.

### 2.10 Quantification and statistical analysis

The median fluorescence intensity (MFI) of NADH and FAD was utilized for all experiments. The statistical analysis was performed using GraphPad Prism 9 (GraphPad Software, San Diego, CA, USA). Data containing two groups were analyzed using a t-test. The data with more than two group data comparisons was either one-way or two-way ANOVA RM with Bonferroni or Turkey's multiple comparisons test. Values are expressed as mean ± s.e.m. Mitochondrial spare capacity from Seahorse measurements was calculated automatically by WAVE software using the formula: (OCR_FCCP42min_ + OCR_FCCP48min_ + OCR_FCCP56min_/3)/(OCR_basal0min_ + OCR_basal8min_ + OCR_basal16min_/3)∗100]. Mitochondrial spare capacity based on NAD(P)H and FAD measurements was calculated as basal fluorescence intensity (FI)/FI after FCCP treatment × 100 [(FI_basal0min_)/FI after FCCP treatment (FI_FCCP28min_ + FI_FCCP36min_/2)∗100].

## 3 Results

### 3.1 Benchmarking the metabolic measurement of the optical redox ratio by flow cytometry

**Figure 1:**
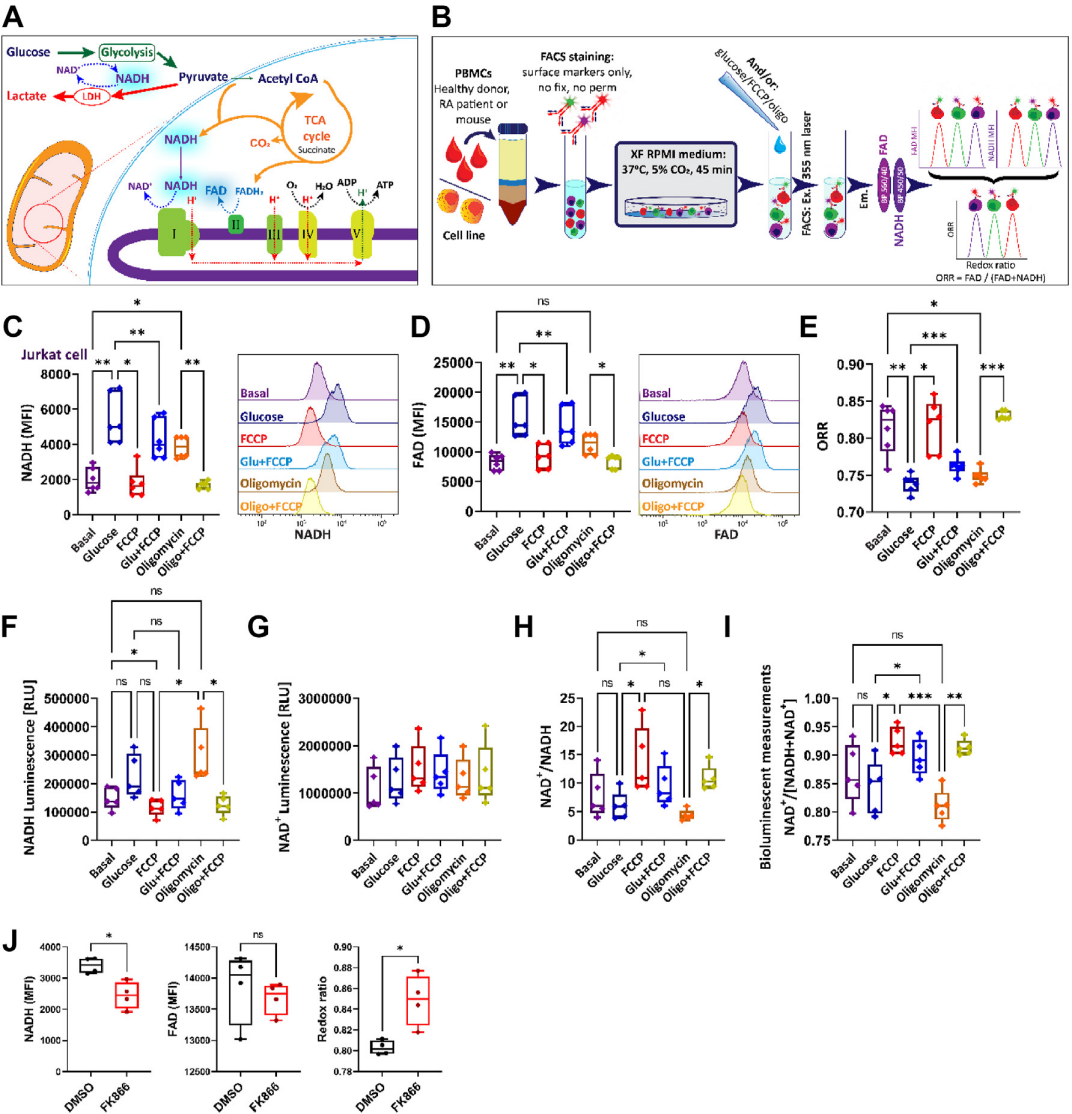
**Changes in the optical redox ratio and NADH//NAD^+^ in different environmental conditions**. A. Diagram depicting the metabolic pathways of glycolysis and cellular respiration, highlighting the involvement of nicotine adenine nucleotide (NAD) and flavine adenine nucleotide (FAD). Included in this schematic is the lactate dehydrogenase (LDH) enzyme and the number I–V represents the mitochondrial respiratory complexes I–V. B. Principal experimental setup to measure the optical redox ratio in complex cell populations under dynamic environmental conditions by flow cytometry. C, D. Human Jurkat T cells were equilibrated in Seahorse XF RPMI medium (glucose-free). At defined time points, glucose (Glu), Carbonyl cyanide-p-trifluoromethoxyphenylhydrazone (FCCP), Oligomycin or combinations thereof were added and NAD(H) (**C**) as well as FAD (**D**) fluorescence (fluorescence intensity, FI) were measured by flow cytometry. One-way ANOVA repeated measures, F = 22.65 (**C**); F = 20.96 (D), ∗p = 0.0117–0.0181, ∗∗p = 0.0020–0.0071, (Turkey multiple comparison test), n = 6, values are means ± SEM. E. The redox ratio (ORR) of Jurkat T cells from the experiment shown in c and d was calculated. One-way ANOVA repeated measures, F = 23.73, ∗p = 0.0230–0.0259, ∗∗p = 0.0030, and ∗∗∗p = 0.0001–0.0007 (Turkey multiple comparison test), n = 6, values are means ± SEM. F-I. Jurkat T cells were placed in glucose-free medium. Glucose, Carbonyl cyanide-p-trifluoromethoxyphenylhydrazone (FCCP) and Oligomycin were added in different combinations, cells were lysed and NAD+ and NADH were quantified enzymatically.F. NADH abundance, G. NAD+ abundance, H. NAD+/NADH ratio, I. NAD+ abundance relative to total NAD+ + NAD(H). Each dot represents one experiment. One-way ANOVA repeated measures, ∗p = 0.0142–0.0436, ∗∗p = 0.0014, ∗∗∗p < 0.0001 (Turkey multiple comparison test), n = 5, values are means ± SEM. J. The Jurkat cells were treated with 1 μM FK866 or DMSO for 60 min after the starvation and the NADH and FAD fluorescence was measured by flow cytometry. NADH and FAD median fluorescence intensities (MFI) were used to quantify the ORR. The statistics were performed with a Paired t-test. ∗p = 0.0302–0.0366, n = 4 stimulations. Values are mean ± SEM.

To study the dynamic regulation of metabolism and the metabolic activity of complex cell populations in real time we made use of the label-free cell intrinsic endogenous fluorescence of NAD(P)H and FAD. NADH is generated during glycolysis and in the Krebs cycle, while FAD is a co-enzyme of succinate dehydrogenase and an intermediate electron acceptor [14,15] (Figure 1A). NAD(P)H and FAD exhibit peak fluorescence at 450 nm and 525 nm, respectively, after excitation at 335 nm [1,16]. By choosing compatible filter settings, the simultaneous determination of the ORR in different cell types within mixed cell populations should be possible (Figure 1B). Specifically, an increase in flux through biosynthetic pathways without a proportional ATP demand, such as acute glucose availability, will cause an increase in NAD(P)H fluorescence and a decrease in the ORR (Fgure 1a). To study first the dynamic regulation of metabolism by flow cytometry, we started with the human T cell lymphoma line Jurkat [29]. These cells were placed into glucose- and serum-free medium. Glucose was added and NAD(P)H fluorescence was measured after defined time points when the increase in NAD(P)H fluorescence has reached a plateau (Figure 1C; Figure 1a and b). Inspired by work on NAD(P)H fluorescence imaging [18,19,22] and NAD(P)H fluorescence lifetime imaging (FLIM) [30] we attempted to determine whether NAD(P)H is used in respiration by applying the protonophore carbonyl cyanide m-chlorophenyl hydrazine (FCCP). Glucose elicited an increase in NAD(P)H and FAD fluorescence (Figure 1C). FCCP reduced NAD(P)H and FAD fluorescence only marginally under basal conditions. However, the NAD(P)H fluorescence was reduced by ∼30% when glucose and FCCP were added simultaneously, suggesting that a fraction of newly generated NAD(P)H enters the electron transport chain (ETC). When Oligomycin was added to cells in basal medium, NAD(P)H fluorescence increased while this effect was less visible for FAD (Figure 1C,D). The NADH fluorescence that was elevated by Oligomycin exposure returned to baseline completely when FCCP was added (Figure 1C), suggesting that it was used in the ETC. FAD behaved similarly but with less inclination (Figure 1D). Although the changes in NAD(P)H and FAD fluorescence correspond to expected changes in their actual amounts, other processes than the ETC can use NAD(P)H and FAD [[31], [32], [33]]. To consequently normalize our measurements, we calculated the optical redox ratio (ORR) of FAD/[NADH + FAD]. The ORR will typically maintain a normal distribution, produce values between 0 and 1 and is an established and standardized parameter [1]. The ORR of Jurkat cells decreased after glucose addition and was only mildly elevated after FCCP treatment under basal or glucose-rich conditions, as expected. Oligomycin diminished the ORR while additional FCCP reverted this decrease (Figure 1E), fitting to data in other cell types [30].

To confirm these data, we measured NADH and NAD^+^ enzymatically with commercial assays (Figure 1F–I), corroborating that FCCP uncouples respiration and depletes mitochondria from NAD(P)H by increasing NAD^+^ [34] (Figure 1G,H; not significant). Moreover, similar to the changes detected by flow cytometry, glucose and Oligomycin elevated NADH while FCCP reverted these changes (Figure 1F). Hence, we calculated the relative abundance of NAD^+^ via the ratio of NAD^+^/[NAD^+^+NADH] (Figure 1I), which has been shown to correlate strongly with the ORR in several cell types [18,22]. Indeed, both methods correlated well (Figure 1E,I): Glucose reduced the ORR and the relative NAD^+^ abundance, but only the change in the ORR reached statistical significance.

FCCP alone had no significant effect but it elevated the ORR and relative NAD^+^ abundance in Glucose-treated cells. Oligomycin lowered both ORR and the relative NAD^+^ abundance, however, this was more significant with the optical method. Simultaneous Oligomycin and FCCP treatment reverted the Oligomycin-induced decline in both experimental setups robustly.

Hence, both methods produce similar results. It appears that the variation of the flow cytometry measurements is generally lower, except for FCCP treatment, and the glucose-elicited changes are more prominent when measured by flow cytometry. To validate our analysis we addressed another metabolic pathway. Replenishment of NAD^+^ is essential to regenerate glycolytic and mitochondrial capacity by enabling the formation of NAD(P)H. Thus, depletion of NAD^+^ should lower NAD(P)H abundance and, consequently, elevate the ORR. The intracellular form of the enzyme Nicotinamide phosphoribosyltransferase (NAmPRTase or NAMPT) is the rate-limiting enzyme in the NAD^+^ salvage pathway. NAMPT converts nicotinamide to nicotinamide mononucleotide. This step is responsible for most of the NAD^+^ formation in mammals [35]. Inhibitors of NAMPT are considered for cancer treatment [36]. Thus, we treated Jurkat T cells with the NAMPT inhibitor FK866 [36]. In line with the predictions, NAMPT inhibition by FK866 reduced NADH fluorescence while not affecting FAD fluorescence, thereby, elevating the ORR (Figure 1J). We conclude that label-free flow cytometry of NADH and FAD fluorescence can depict acute metabolic changes accurately and appears to be more stable than enzymatic measurements.

### 3.2 Side by side comparison of dynamic single-cell metabolic flow cytometry to extracellular flux analysis

**Figure 2:**
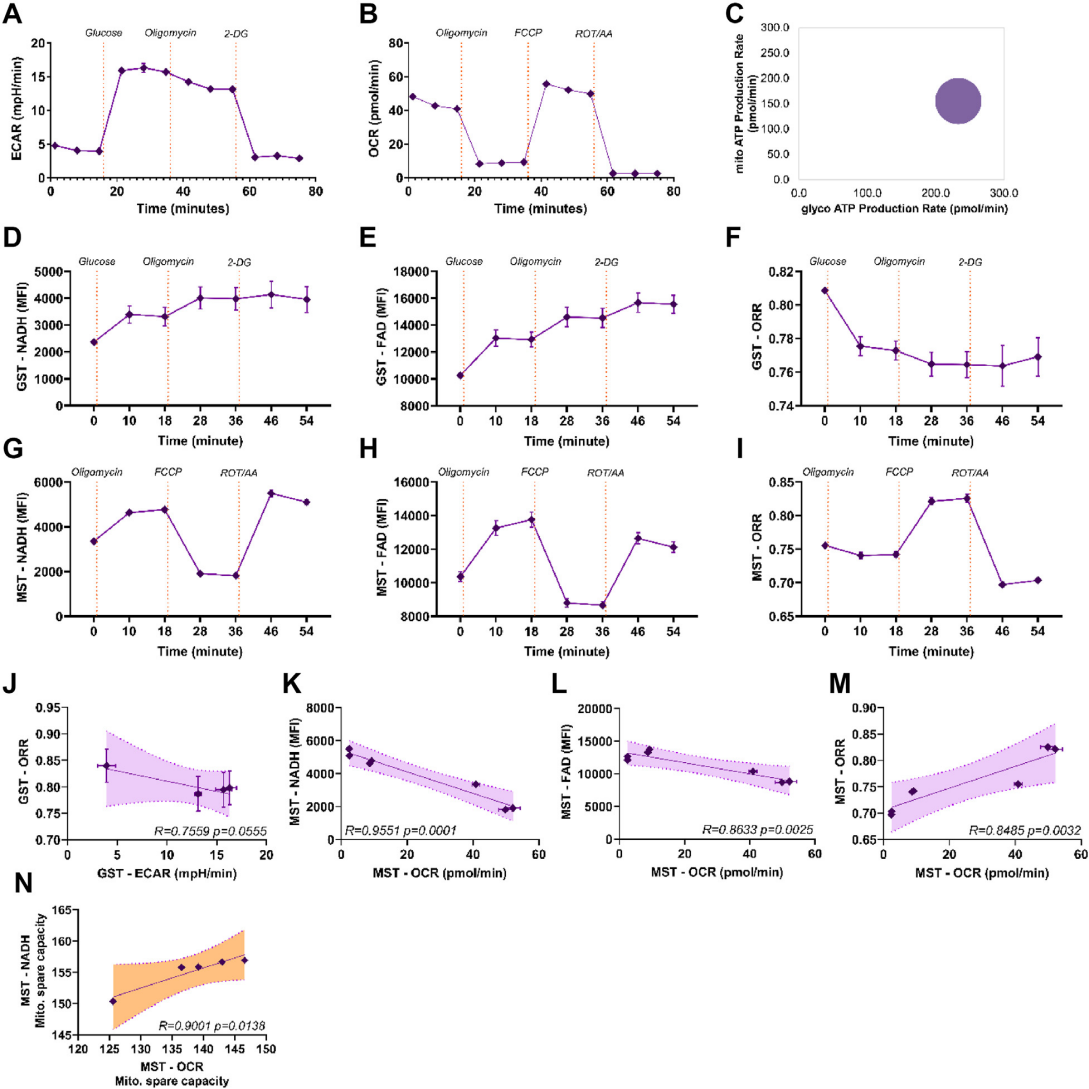
**Analysis of glycolytic and respiratory capacities of Jurkat cells via Seahorse and optical redox ratio**. A. Jurkat T cells were placed in a glucose-free medium, glucose, oligomycin and 2-Deoxyglucose (2-DG) were added at indicated time points and the extracellular acidification rate (ECAR) was measured by Seahorse Glyco Stress Test (GST). The experimental data is presented as mean ± SEM. n = 4. B. Jurkat T cells were placed in Mito Stress Test medium, then oligomycin, carbonyl cyanide m-chlorophenyl hydrazine (FCCP), Rotenone/Antimycin (ROT/AA) were added at the indicated time points and the oxygen consumption rate (OCR) was measured by Seahorse XF^e^96 machine with Seahorse wave Mito Stress Test (MST) protocol. The experimental data is presented as mean ± SEM. n = 4. C. The energetic profile of Jurkat cells was calculated via ECAR and OCR measurements by WAVE software. D, E. Jurkat T cells were placed in a glucose-free medium, then glucose, oligomycin, and 2-Deoxyglucose (2-DG) were added at indicated time points and NADH as well as FAD fluorescence (FI) were measured by flow cytometry. The experimental data is presented as mean ± SEM. n = 7. F and I. The GST and MST redox ratio (ORR) of the Jurkat T cell was calculated with the NADH and FAD values from experiments d, e, g, and h. The experimental data is presented as mean ± SEM. n = 7. J. Linear regression analysis of GST-ORR of Jurkat call correlated to ECAR of Seahorse GST data of the same Jurkat cell samples with a R-squared value of 0.7559, p = 0.0555, n = 4–6. K-M. The linear regression analysis shows that the MST NADH, FAD, and ORR of Jurkat call are directly correlated to the OCR of Seahorse MST data of the same Jurkat cell samples with a R-squared value of 0.9551, 0.8633, and 0.8485 respectively (p = 0.0001–0.0032, n = 4–6). N. The mitochondrial spare capacity data of the Seahorse of the Jurkat cell were compared with the mitochondrial spare capacity data obtained from NADH fluorescence. Linear regression analysis reveals that they are directly correlated with a R-squared value of 0.9001 (p = 0.0138, n = 4–6). J-N. Each data point represents the values of the cellular respiration (OCR or ECAR) with/without inhibitors measured with the Seahorse compared to the dynamic measurement with the flow cytometry (NADH or FAD fluorescence) at the same time point. The purple line is the general linear fit with all the data points. The dotted line indicates a 99% confidence band.

To validate these data, we performed a Seahorse Glyco Stress Test (GST) experiment by extracellular flux analysis (Figure 2A). In such experiments, similar to the previous experiments (Figure 1, Fig. S1), the cells are kept in a glucose-free basal medium, glucose is added and if cells are glycolytic, ECAR as well as NADH (Figure 1C, Fig. S1) will increase. Afterwards, Oligomycin is added. If cells perform considerable respiration, Oligomycin will drive the cells into anaerobic glycolysis and ECAR will increase even more. The increase of ECAR will be reverted by 2-Deoxyglucose (2-DG) treatment. The GST revealed that Jurkat cells increase ECAR after glucose addition (Figure 2A). Oligomycin had a small additional effect (Figure 2A), suggesting that under the given conditions, Jurkat cells have little excessive glycolytic capacity. We also performed a Seahorse Mito Stress Test (MST) experiment. There, cells respire basally in full medium. Oligomycin stops O_2_ consumption, which will in turn increase to its maximum after uncoupling via FCCP treatment, and cease again after the abolishment of complex I and III activity by adding Rotenone/Antimycin A. The MST showed that the Jurkat cells under our conditions respire at ∼50 pMol/min/cell with slight mitochondrial spare capacity (Figure 2B). The ECAR/OCR profile classified them as glycolytic (Figure 2C). Next, we used the “Seahorse” protocols to measure ORR. In a simulated GST, there was a glucose-mediated increase in NAD(P)H and FAD fluorescence similar to ECAR (Figure 2D,E) and a small additional increase after Oligomycin addition. In contrast to ECAR, neither NAD(P)H nor FAD decreased after 2-DG addition in the depicted time frame. Likely, even after 2-DG addition, NAD(P)H and FAD are maintained or generated downstream of LDH activity, reflecting mitochondrial spare capacity based on previous pyruvate import into mitochondria. Notwithstanding, as expected, the ORR declined upon glucose and Oligomycin treatment (Figure 2F). In the MST conditions, the time-dependent changes of NAD(P)H and FAD fluorescence proceeded reciprocally compared to the pO_2_ measurements of the Seahorse (Figure 2G,H). In contrast, the ORR changed proportional to pO_2_, except that the Oligomycin-induced decline was smaller (Figure 2I). As we know that NAD(P)H and FAD fluorescence are not calibrated, we wondered whether they correlate with the results of extracellular flux analysis. Therefore we performed regression analysis. While the ORR during the GST correlated hardly with the ECAR data (Figure 2A,F,J), the ORR correlated strongly with OCR as well as with NAD(P)H fluorescence (Figure 2K,M). FAD fluorescence correlated as well (Figure 2L). Moreover, mitochondrial spare capacities as calculated by extracellular flux analysis and by the flow cytometry method correlate also strongly (Figure 2N). We conclude that measuring both NAD(P)H and FAD as ORR via sequential application of metabolic inhibitors by flow cytometry can provide information that is related and complementary to OCR and ECAR.

### 3.3 Employing genetic alterations of metabolism to calibrate redox ratios in primary mouse B cells

**Figure 3:**
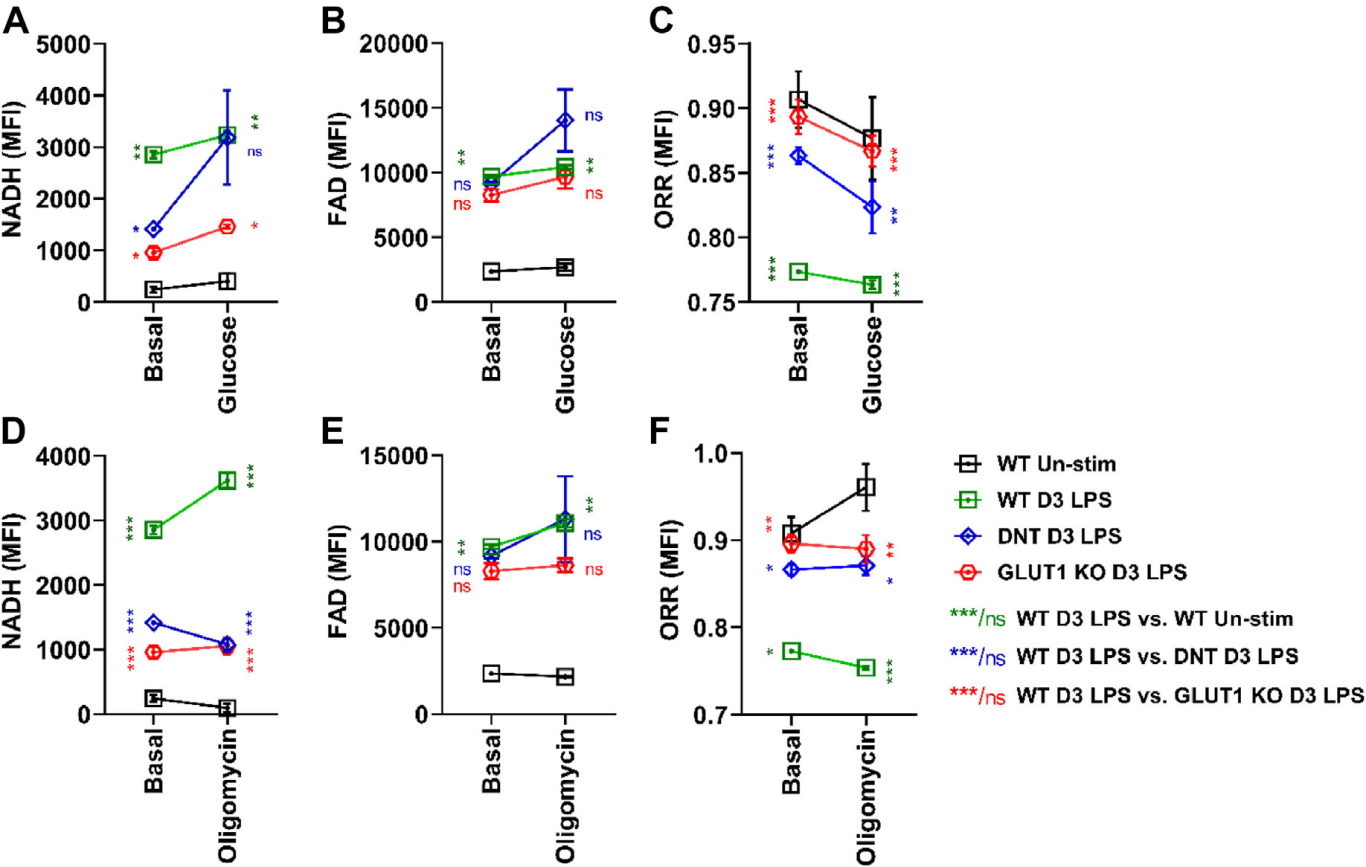
**Calibration of NADH fluorescence with mutant primary mouse B cells**. A, B, D, and E. B cells from DNT (CD23Cre x dominant-negative TWINKLE K320E) or GLUT1KO (*glut1*^fl/fl^ x CD23Cre) mice were left unstimulated or were stimulated for 3 days with lipopolysaccharide, placed in glucose-free medium and glucose or Oligomycin were added to the sample. Afterwards, NADH and FAD fluorescence (FI) were measured by flow cytometry. Cells were unstained and gated according to Fig. S2 (lymphocytes and live cells). For the analysis of dominant-negative TWINKLE K320E, GFP^+^ populations were used. Two-way ANOVA repeated measures, ∗p = 0.01–04, ∗∗p = 0.002–0.007, and ∗∗∗p ≤ 0.001 (Tukey's multiple comparison test), n = 3 independent experiments, values are means ± SEM. C and F. The redox ratio FAD/(FAD + NADH) of stimulated B cells from the experiments shown in A, B, D, and E. Two-way ANOVA repeated measures, ∗p = 0.01–04, ∗∗p = 0.002–0.009, and ∗∗∗p ≤ 0.001 (Tukey's multiple comparison test), n = 3, values are means ± SEM.

To identify physiological correlates of altered ORR and to further validate the method, we proceeded with primary, unstained mouse B cells (Fig. S2). We measured the ORR of lipopolysaccharide (LPS) activated primary wildtype (WT, CD23Cre) B cells, of B cells where the floxed gene encoding glucose transporter GLUT1 is deleted via CD23 mediated Cre expression (GLUT1KO) [37], and of B cells where OxPhos is genetically reduced by CD23Cre-controlled expression of a dominant-negative version of the mitochondrial helicase Twinkle linked to IRES-GFP (TwinkleK320E; DNT; [6]). MtDNA encodes essential subunits of mitochondrial complexes I, III, IV and V while complex II (SDH) is encoded entirely in the nucleus [38]. Hence, TwinkleK320E/DNT-expressing B cells show defective replication of mtDNA, reduced OxPhos and a stalled TCA cycle, but increased glycolysis [6]. In line with published data [23], LPS activated WT B cells had a stronger NAD(P)H fluorescence than resting B cells. Almost comparable to non-activated B cells, LPS activated GLUT1KO and DNT B cells showed less NAD(P)H fluorescence than WT B cells while the FAD fluorescence was similar (Figure 3A). To induce acute formation of NAD(P)H and FAD, we added glucose. In response to glucose, DNT B cells increased NAD(P)H but also FAD fluorescence by trend more than WT or GLUT1KO B cells, suggesting that they have an increased glycolytic capacity, confirming previous data obtained with Seahorse analysis [6]. The ORR was higher in resting TwinkleK320E/DNT and GLUT1KO B cells than in activated WT B cells (Figure 3C) but it appeared to drop more in glucose-stimulated DNT B cells than WT B cells (Figure 3C). When ATP synthase was inhibited with Oligomycin, only activated WT B cells increased NAD(P)H fluorescence, suggesting that these cells oxidize NADH basally in the ETC (Figure 3D). Accordingly, the low respiration of DNT B cells was reflected in their unresponsiveness. In confirmation of mitochondrial defects, also GLUT1KO B cells did not react [37] (Figure 3D). Hence, the ORR of resting and activated DNT and GLUT1KO B cells is higher than that of glucose-treated and activated WT B cells (Figure 3F). To exclude artifacts in DNT B cell cultures elicited by GFP expression we expressed GFP alone in LPS activated B cells (Fig. S2c), revealing no effect. In the absence of glucose, the ORR is higher in cells with primary [6] and secondary [37] OxPhos defects. With regard to NAD(P)H, ATP synthase inhibition had opposite effects in OxPhos-competent vs. non-competent cells (Figure 3D). Overall, these experiments demonstrate that metabolic defects alter the ORR prominently in primary cells, re-confirming the applicability of ORR as a measure to determine the metabolic state at single cell level.

### 3.4 NAD(P)H and FAD fluorescence measurements of surface-stained primary B cells

**Figure 4:**
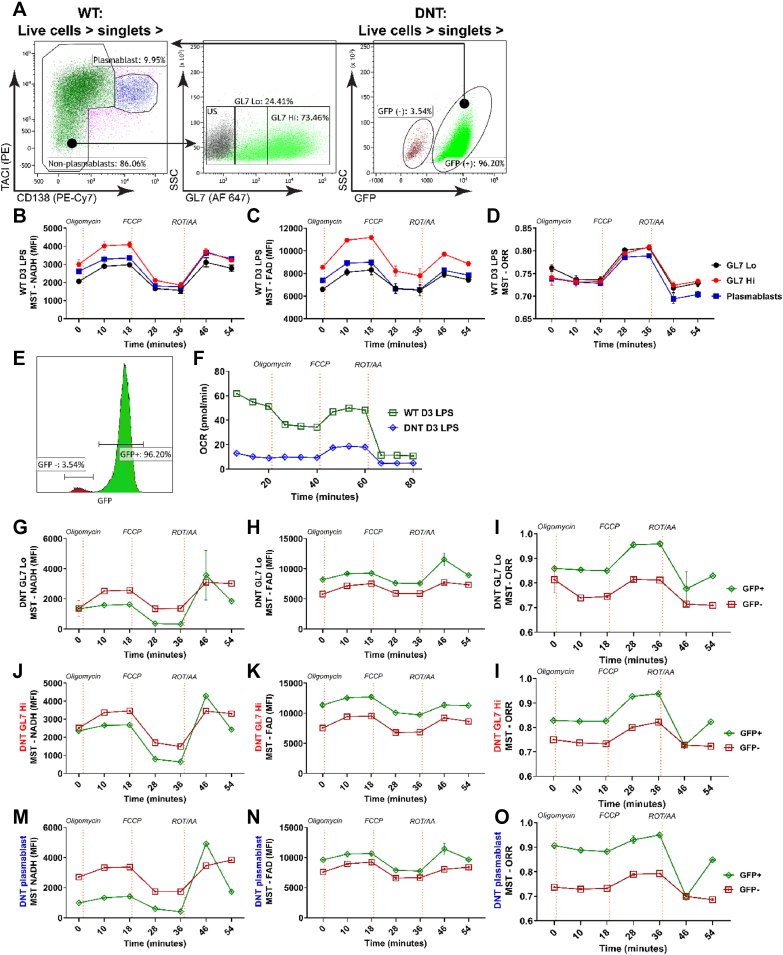
**Simultaneous determination of optical redox ratios in different B cell populations**. A. B cells from wildtype mice (C57Bl/6) were stimulated for 3 days with lipopolysaccharide, surface-stained as indicated, and analyzed by flow cytometry. In the representative dot plot, the plasmablasts were identified as TACI^+^ and CD138^+^. The non-plasmablasts were gated further to analyze GL7^hi^ and GL7^Lo^ (green) populations. US: marked black populations are unstained. B, C. B cells from wildtype mice (C57Bl/6) were stimulated for 3 days with lipopolysaccharide, surface-stained as indicated in a. and placed in Mito Stress Test medium. Oligomycin, carbonyl cyanide m-chlorophenyl hydrazine (FCCP), and Rotenone/Antimycin were added at indicated time points and NADH and FAD fluorescence (FI) were measured by flow cytometry. Values are means ± SEM. n = 2 independent cultures. D. The redox ratio [FAD]/([FAD]+[NAD(H)]) of stimulated B cells from the experiments shown in B, C; Values are means ± SEM. n = 2 independent cultures. E. B cells from CD23Cre and CD23Cre x TwinkleK320E (DNT) mice were stimulated for 3 days with lipopolysaccharide and surface stained. GFP^−^ and GFP^+^ cells of the indicated populations are depicted and further gated for the plasmablasts, GL7^Lo^, and GL7^Hi^. F. B cells from CD23Cre, CD23Cre x *glut1*^fl/fl^ mice and CD23Cre x TwinkleK320E (DNT) mice were stimulated for 3 days with lipopolysaccharide and placed in Mito Stress Test medium. Oligomycin, carbonyl cyanide m-chlorophenyl hydrazine (FCCP), and Rotenone/Antimycin were added at indicated time points and the oxygen consumption rate (OCR) was measured by Seahorse Mito Stress Test (MST). Values are means ± SEM. n = 2 independent cultures. G, H. B cells from CD23Cre, CD23Cre x *glut1*^fl/fl^ mice and CD23Cre x TwinkleK320E (DNT) mice were stimulated for 3 days with lipopolysaccharide, surface stained and placed in Mito Stress Test medium. Oligomycin, FCCP, and Rotenone/Antimycin were added at indicated time points and NADH/FAD fluorescence of GL7^high^ cells were measured by flow cytometry. Values are means ± SEM. n = 2. I. The redox ratio [FAD]/([FAD]+[NAD(H)]) of stimulated B cells from the experiments shown in G, H; Values are means ± SEM. n = 2 independent cultures.

Next, we correlated extracellular flux analysis with ORR in primary murine WT and DNT B cells (gating strategy in Figs. S2 and 4a). First, we analyzed bulk WT B cells. Albeit there were statistically significant correlations between OCR/ORR, OCR/FAD and OCR/NAD(P)H in a simulated MST, the correlations were less significant than with Jurkat cells (Fig. S3, Figure 2K–N). We speculated that this could be due to a heterogeneous population, with different kinetics and quantitative reactions of subsets. In fact, LPS cultures contain activated (TACI^high^GL7^high^), less activated (GL7^low^) and differentiated cells (plasmablasts, TACI^+^CD138^+^) [6,39] (Figure 4A). Moreover, DNT B cell cultures contain non-recombined GFP^−^ cells with an uncompromised capacity to perform OxPhos (Figure 4A). Therefore, we measured the ORR of different surface-stained populations after the sequential addition of inhibitors, as in a MST (Figure 4B). Importantly, surface staining with fluorescently labeled antibodies did not change NAD(P)H or FAD fluorescence (Fig. S2b). WT GL7^high^ cells showed more basal NAD(P)H and FAD and higher FAD after Oligomycin addition compared to GL7^low^ cells. TACI^+^CD138^+^ plasmablasts were intermediate (Figure 4B). These data suggest a heightened NAD/NAD(P)H and FADH_2_/FAD turnover in GL7^high^ cells. Nevertheless, the ORR was remarkably constant, suggesting that the various differentiation stages balance the reported changes in nutrient uptake [40] efficiently to maintain energy and redox balance (Figure 4D). We conclude that LPS cultures contain metabolically distinct subsets. This may bias bulk measurements, underlining the importance to perform metabolic profiling at the single-cell level.

To more rigorously test this hypothesis, we next focused on the TwinkleK320E/DNT B cells, corroborating defective OxPhos and a proportion of GFP^−^ cells [6] in 3 d LPS bulk cultures (Figure 4E,F). We then split the GL7^low^, GL7^high^ and TACI^+^CD138^+^ populations in GFP^+^ and GFP^−^ cells (Figure 4G–O). In all conditions and populations, GFP^+^ cells revealed less NAD(P)H fluorescence, more FAD fluorescence and a high ORR. Rotenone/AntimycinA elicited transient overshooting reactions in DNT B cells, indicating a dysbalanced NAD(P)H/FAD homeostasis. The ORR of the GFP^−^ cells (stained with anti CD138, TACI and GL7 markers, gated as live and GFP^−^) correlated almost perfectly with those of unstained bulk WT B cells in the same conditions (Fig. S3, d-f). To conclude, (I) the determination of NAD(P)H/FAD fluorescence of surface stained cells by flow cytometry is possible without adverse effects of antibody surface staining. (II) NAD(P)H and FAD fluorescence are compatible with GFP emission. (III). Genetic manipulation of glycolysis and OxPhos are reflected in the ORR. (IV). The metabolism of different cell populations within one culture can be resolved by real time flow cytometry. Hence, metabolic alterations should be amenable to unbiased flow cytometric determination of the ORR. To substantiate this statement further, we asked whether Glutamine and fatty acids impact the ORR. Both have been implicated in the mitochondrial metabolism of differentiating B cells [40]. To assess the contribution of Glutamine we cultured LPS activated B cells in standard (2 mM) or reduced (200 μM) concentrations of Glutamine (Fig. S4a). In line with the expectations, activated B cells (TACI^+^) showed a lower ORR in 200 μM Glutamine but were able to increase the ORR in response to acute Glutamine availability (Fig. S4b). Plasmablasts (CD138^+^TACI^+^) showed surprisingly overall a higher ORR in 200 μM than in 2 mM Glutamine. However, under neither condition did they increase the ORR upon acute Glutamine exposure (Fig. S4b). In contrast, both subsets as well as Jurkat T cells responded equally to Palmitate with an increase of the ORR (Figs. S4c and d). Together, these notions support previous data hinting at metabolic alterations during the different stages of plasma cell development [40] and open up the possibility of assessing amino acid and fatty acid metabolism by flow cytometry.

### 3.5 Human memory B cells have higher metabolic capacities than naïve B cells

**Figure 5:**
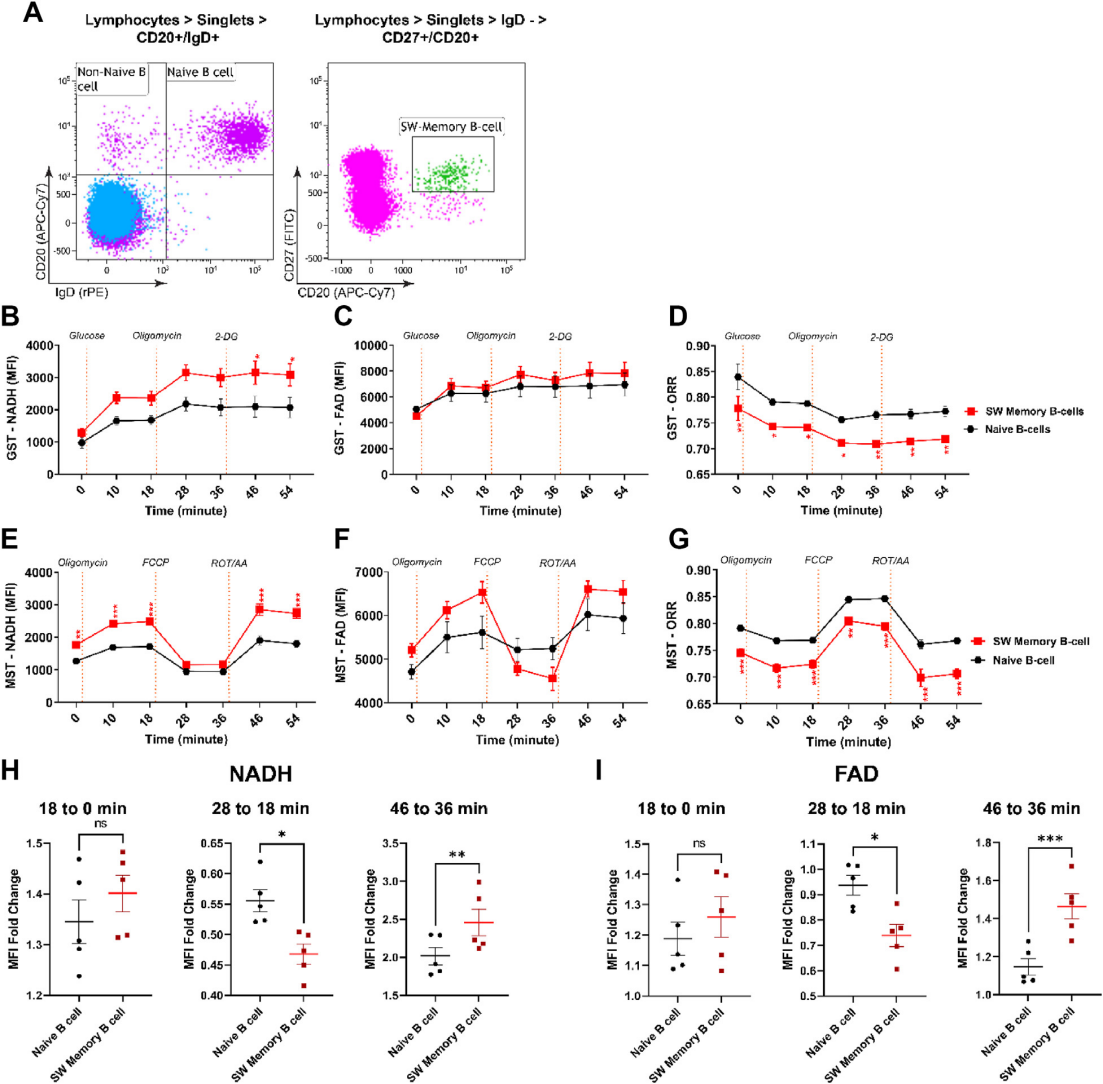
**Simultaneous determination of optical redox ratios in different populations of human peripheral blood mononuclear cells**. Peripheral blood mononuclear cells (PBMC) from a healthy donor were surface-stained as indicated and analyzed by flow cytometry. In the representative dot plot, CD20^+^, IgD^+^ cells are identified as naïve B-cells and IgD^−^, CD27^+^, and CD20^+^ cells are Switched (SW) memory B-cells. B, C. PBMC from HD were surface-stained as indicated in (A) and placed in a glucose-free medium. Glucose, Oligomycin and 2-Deoxyglucose (2-DG) were added at indicated time points and NADH as well as FAD fluorescence (FI) was measured by flow cytometry. Two-way ANOVA repeated measures, ∗p = 0.03–0.04 (Bonferroni multiple comparison test), n = 5 different donors, values are means ± SEM. D. The redox ratio [FAD]/([FAD]+[NAD(H)]) of PBMC from the experiment shown in b, c. Two-way ANOVA repeated measures, ∗p = 0.0189–0.0315, ∗∗p = 0.0012–0.0075 (Bonferroni multiple comparison test), n = 5 different donors, values are means ± SEM. E, F. PBMC from HD were surface-stained as indicated in (A) and placed in the Mito Stress Test medium. Oligomycin, carbonyl cyanide m-chlorophenyl hydrazine (FCCP), and Rotenone/Antimycin were added at indicated time points and NADH and FAD fluorescence were measured by flow cytometry. Two-way ANOVA repeated measures, ∗∗p = 0.003, ∗∗∗p ≤ 0.001 (Bonferroni multiple comparison test), n = 5 different donors, values are means ± SEM. G. The redox ratio [FAD]/([FAD]+[NAD(H)]) of PBMC from the experiment shown in E, F. Two-way ANOVA repeated measures, ∗∗p = 0.002, ∗∗∗p ≤ 0.001 (Bonferroni multiple comparison test), n = 5 different donors, values are means ± SEM. H. The relative change of NADH was calculated between the 0–18, 18–28, and 36–46 min time points and was analyzed using a paired t-test. ∗p = 0.01, ∗∗p = 0.003 and n = 5 different donors. I. The relative change of FAD was calculated between the 0–18, 18–28, and 36–46 min time points and was analyzed using a paired t-test. ∗p = 0.01, ∗∗∗p < 0.001 and n = 5 different donors.

**Figure 6:**
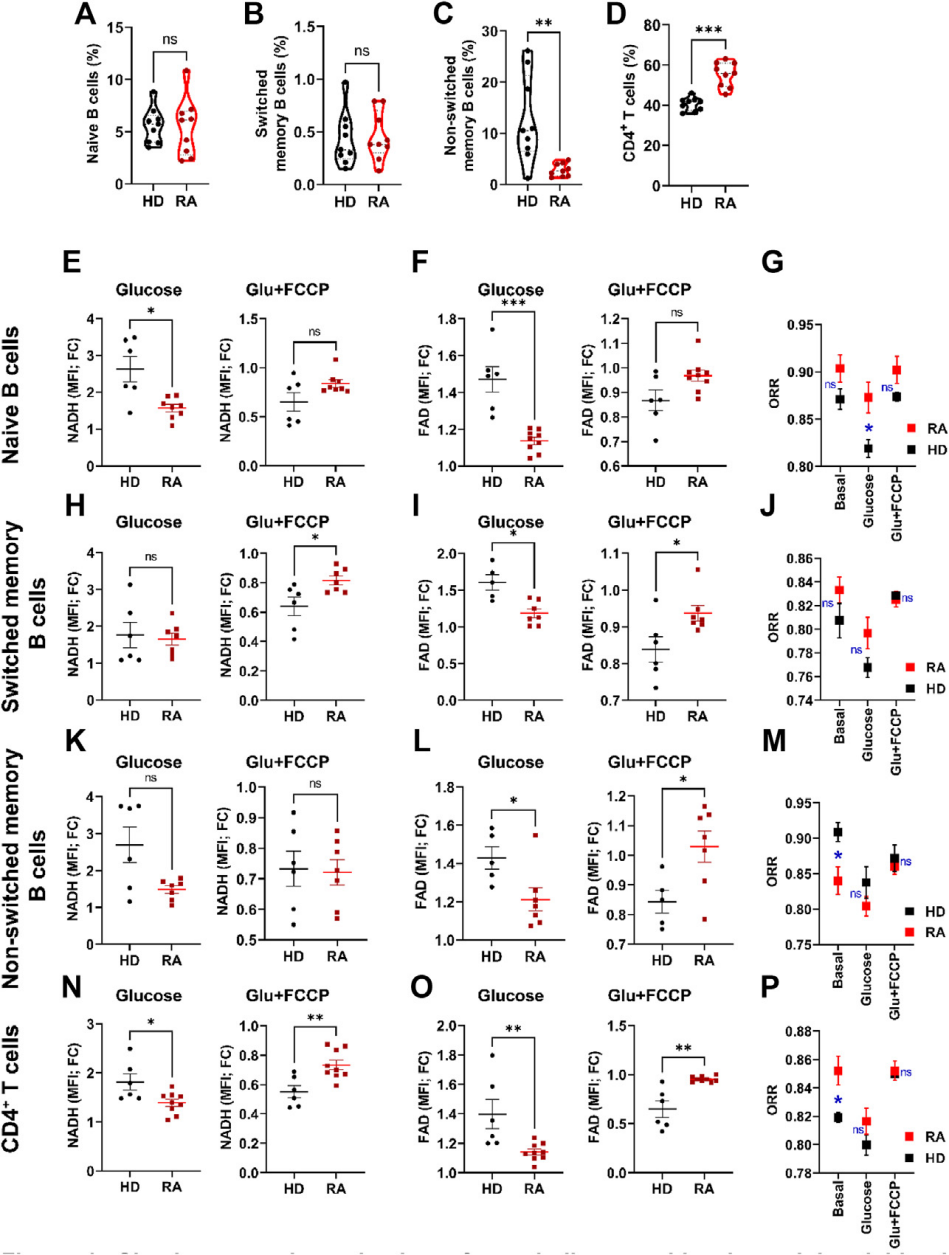
**Simultaneous determination of metabolic capacities in peripheral blood mononuclear cells of rheumatoid arthritis patients**. Determination of frequencies of naïve B cells (A), switched (B) and Non-switched (C) memory B cells as well as CD4^+^ T cells (D) from Peripheral blood mononuclear cells (PBMC) from healthy donors and rheumatoid arthritis (RA) patients. Unpaired t-test (two-tailed). ∗∗p = 0.0035, ∗∗∗p < 0.001 and n = 9 different donors. The experimental data is presented as mean ± SEM. E and F. PBMC from HD and RA patients were surface-stained and placed in a glucose-free medium. Glucose and Glucose + FCCP were added and NADH as well as FAD fluorescence from naïve B cells were measured by flow cytometry. The relative changes were calculated, unpaired t-test, ∗p = 0.01, ∗∗∗p < 0.00, n = 6–8 different donors, values are mean ± SEM. G. The redox ratio [FAD]/([FAD]+[NAD(H)]) of PBMC from the experiment shown in E, F. Two-way ANOVA, ∗p = 0.03, (Turkey multiple comparison tests), n = 6–8 different donors, values are mean ± SEM H and I. PBMC from HD and RA patients were surface-stained and placed in a glucose-free medium. Glucose and Glucose + FCCP were added and NADH as well as FAD fluorescence from switched memory B-cells were measured by flow cytometry. The relative changes were calculated, unpaired t-test, ∗p = 0.01–0.03, n = 6–7 different donors, values are means ± SEM. J. The redox ratio [FAD]/([FAD]+[NAD(H)]) of PBMC from the experiment shown in H, I. Two-way ANOVA, (Turkey multiple comparison tests), n = 6–7 different donors, values are mean ± SEM K and L. PBMC from HD and RA patients were surface-stained and placed in a glucose-free medium. Glucose and Glucose + FCCP were added and NADH as well as FAD fluorescence from non-switched memory B cells were measured by flow cytometry. The relative changes were calculated, unpaired t-test, ∗p = 0.03, n = 5–7 different donors, values are means ± SEM. M. The redox ratio [FAD]/([FAD]+[NAD(H)]) of PBMC from the experiment shown in K, L. Two-way ANOVA, ∗p = 0.0471, (Turkey multiple comparison tests), n = 5–7 different donors, values are mean ± SEM. N and O. PBMC from HD and RA patients were surface-stained and placed in a glucose-free medium. Glucose and Glucose + FCCP were added and NADH as well as FAD fluorescence from CD4^+^ T cells were measured by flow cytometry. The relative changes were calculated, unpaired t-test, ∗p = 0.04, ∗∗p = 0.002–0.008, n = 6–9 different donors, values are means ± SEM. P. The redox ratio [FAD]/([FAD]+[NAD(H)]) of PBMC from the experiment shown in N, O. Two-way ANOVA, ∗p = 0.02, (Turkey multiple comparison tests), n = 6–9 different donors, values are mean ± SEM.

Having established single-cell measurements of the ORR by flow cytometry, we tested whether we can analyze human peripheral blood mononuclear cells (PBMC) in an unbiased manner as suggested earlier [23]. We focused on B cells, because it has been shown that human memory B cell differentiation into plasma cells involves metabolic reprogramming [41]. Here, we discriminated naïve B cells (CD20^+^IgD^+^CD27^-^), non-switched memory B cells (IgD^+^CD20^+^CD27^+^) and switched memory B cells (IgD^−^CD20^+^CD27^+^) [without re-confirming somatic hypermutation 42,43], as well as T helper cells (CD4^+^) by surface marker expression (Figure 5A; Figs. S5a and S6a). Similar to mouse B cells, surface staining of PBMC with fluorescently labeled antibodies had no effect on NAD(P)H or FAD fluorescence (Fig. S5b). We first asked whether naïve human B and T cells as well as memory B cells within the same healthy donor (HD) sample exhibit metabolic alterations in response to Glucose (Figure 5B–D, Fig. S5). Figure 5 shows the comparison of naïve and switched memory B cells; non-switched memory B cells and T cells are included in Figure 6. Remarkably, the ORR was over the complete experiment lowest in switched memory cells and highest in naïve B cells and T cells; non-switched memory B cells were in between (Figure 5D; Fig. S6). Switched memory B cells showed more NAD(P)H fluorescence after Glucose and Oligomycin addition than naïve B cells (Figure 5B) while FAD behaved similarly between naïve and both memory subsets (Figure 5C; S6). CD4^+^ T cells reacted like naïve B cells (Figure 5B,C; S6). Next, we addressed mitochondrial function by adding sequentially Oligomycin, FCCP and Rotenone/Antimycin A (Figure 5E; see Figure 2). Switched memory B cells displayed more NAD(P)H fluorescence than naïve B cells, except for the FCCP-treated samples, while the relative increase after Oligomycin addition was unchanged (Figure 5E, H). In Oligomycin-treated switched memory B cells, FCCP elicited a sharp drop in NAD(P)H fluorescence, which was also much more reverted by Rotenone/Antimycin A treatment in switched B cells than in naïve B cells (Figure 5E, H). Similar results were obtained with T cells but not with non-switched memory B cells (Fig. S6). FAD fluorescence behaved similarly: FCCP hardly reduced FAD fluorescence in naive Oligomycin-treated B cells but there was a strong decline in switched memory B cells and an equally strong reversion by Rotenone/Antimycin A (Figure 5F, I; S6). Very robustly, over the whole experiment and in line with the previous experiments (Figure 5D), the ORR was lowest in switched memory B cells, followed by non-switched memory B cells, naïve B cells and CD4^+^ T cells (Figure 5G, S6). Taken together, switched human memory B cells display larger oscillations of NADH and FAD fluorescence upon metabolic manipulation by mitochondrial uncoupling than naïve B cells.

### 3.6 B lymphocytes from rheumatoid arthritis patients show alterations in glucose metabolism

Next, we asked whether a disease, such as RA, is reflected in lymphocyte metabolism. Both B and T cells contribute to RA. which is associated with antibodies directed against citrullinated peptides (ACPA) [44]. T cells of RA patients often have metabolic defects but information about B cells is sparse [45]. We hypothesized that the ORR of B cells from healthy donors (HD) should differ from those of ACPA-positive RA patients, speculating that autoimmune B lymphocytes are more active. To standardize, we worked with frozen and overnight rested cells. ACPA-positive RA patients (Supplemental Table 1) exhibited reduced frequencies of non-switched memory B cells and increased CD4^+^ cells (Figure 6A–D). To determine their metabolic capacities we stimulated resting cells consecutively with glucose and FCCP. Glucose elicited a stronger increase in NAD(P)H fluorescence in naïve B cells and CD4^+^ cells from HD than from RA donors (Figure 6E,N). When we added FCCP on top of Glucose, the glucose-enhanced NAD(P)H fluorescence decreased more in switched memory B cells and T cells from HD than from RA donors (Figure 6H,N), but not in naïve and non-switched memory B cells (Figure 6E,K). The increment of FAD fluorescence was always higher in cells from HD (Figure 6F,I,L,O) when Glucose was added. Yet, its reciprocal decline after FCCP treatment was pronounced selectively in non-switched and switched B cells as well as T cells from HD than in those of RA patients (Figure 6I,L,O). In consequence, the ORR was lower in naïve B cells from HD upon glucose addition (Figure 6G). Conversely, the ORR was lower in resting CD4^+^ T cells from HD than in RA patients (Figure 6G,P). While the ORR of glucose treated naïve B cells was lower in HD and by trend also in switched memory B cells and T cells, it was reverted more after consecutive FCCP treatment in naïve B cells from HD (p = 0.05), in switched memory B cells (p = 0.03) and by trend also in T cells (Figure 6G,J,P). Interestingly, the ORR was higher in non-switched memory B cells from HD but this difference disappeared after glucose addition, suggesting a better responsiveness to glucose in cells from HD (Figure 6M). Collectively, these data show that (i) there is more glucose induced FAD fluorescence in in naïve, non-switched and switched memory B cells as well as CD4^+^ T cells from HD than in RA donors, (ii) that the glucose-induced FAD is coupled more efficiently to uncoupled respiration in non-switched and switched memory B cells as well as CD4^+^ T cells from HD than in RA donors. In summary, our analyses suggest that RA manifests a metabolic condition where B and T lymphocytes are dampened in glucose mediated pathways.

## 4 Discussion

This report examines the metabolic activity of B and T cells using real-time, non-invasive endogenous fluorescence measurements of NADH/NADPH (short: NAD(P)H) and FAD by flow cytometry. The optical redox ratio (ORR) quantifies the changes in the ratio of oxidized FAD to reduced NAD(P)H in response to the manipulation of anaerobic-like glycolysis and OxPhos. We discuss the relationship between flow cytometric data and extracellular flux analyses that measure oxygen consumption rate (OCR) and extracellular acidification rate (ECAR) in Jurkat T cells. Thereby, we demonstrate the accuracy of flow cytometry in depicting acute metabolic changes and the comparability to extracellular flux analysis, providing complementary information on cellular metabolism Treatment of Jurkat T cells with the NAMPT inhibitor FK866 and genetic manipulation of glycolysis via GLUT1 deletion and mitochondrial respiration in mouse B cells validates our conclusions. Analyzing pathophysiological changes of the ORR in different populations of frozen human PBMC of healthy subjects and RA patients, we reveal that resting switched memory B cells exhibit a lower ORR than naïve B cells, suggesting they are more poised to glycolysis while also being able to reduce NAD(P)H and FAD fluorescence more during uncoupled respiration. Contrastingly, B cells from RA patients appear less susceptible to glucose-induced changes of NADH and FAD fluorescence and they appear to couple glucose-induced FAD less well to mitochondrial respiration. These findings suggest that not only T lymphocytes [45] but also B lymphocytes exhibit metabolic alterations in RA. Identifying the underlying mechanism could identify metabolic vulnerabilities which might be exploited for the development of diagnostic approaches or specific B cell targeting therapies.

Our work relies on the assumption that the NAD(P)H we measure is generated during glycolysis and the Krebs cycle, and that FAD is generated as a coenzyme of the SDH complex. Yet, there are alternative catabolic and anabolic pathways controlling NADH, NADPH and FAD abundance, for instance, the NAD^+^ salvage pathway [35], whose activity was uncovered by our measurements in Jurkat T cells. However, a strong argument for the involvement of Glucose metabolism in NADH homeostasis is the low NADH abundance in activated B cells from B cell-specific GLUT1KO mice [37]. Apoptosis can also affect NADH fluorescence; it has been shown to decrease NADH fluorescence in immune cells, likely via reactive oxygen species (ROS) mediated oxidation [23]. B lymphocytes are capable of producing ROS and H_2_O_2_, and they can activate NADPH-dependent NOX enzymes, which are required for sustained B cell proliferation after B cell receptor stimulation via ROS [46], [47], [48]. However, these NADPH-dependent processes occur after prolonged (2–6 h) stimulation of B lymphocytes via the BCR, a condition that we did not apply. Here, we gated on live cells and even after 35 min of various treatments we could observe increases in NADH, suggesting that our cells were alive. Moreover, we did observe the reversion of the Glucose-mediated increment of NAD(P)H by FCCP via both flow cytometry and enzymatic assays. Because FCCP is a protonophore, it can only act on intact mitochondrial membranes capable of holding the mitochondrial membrane potential (ΔΨm). The hallmark of apoptotic cells are leaky mitochondrial membranes [49]. Consequently, mitochondrial membranes in cells on which FCCP can act by NADH conversion to NAD^+^ should be intact and these cells should not be apoptotic. Therefore, we are confident that the oscillations in NADH fluorescence are not due to apoptosis or other forms of cell death, albeit they may in fact in part also arise from oscillations of NADPH fluorescence which we cannot distinguish [50]. We propose that the fluorescent oscillations of NAD(P)H and FAD we observed here upon acute manipulation of glycolysis and OxPhos in B and T lymphocytes are of metabolic nature.

Measurements of extracellular flux, i.e. changes in ECAR and OCR, have enriched the assessment of cell metabolism. Yet, they rely on pure populations containing high numbers of cells. Simultaneous discriminative metabolic analyses of different cell populations within one sample are impossible with this method. Moreover, a certain threshold number of cells is required for this type of analysis, and this number is sometimes very hard to obtain [51]. Here, we aimed at combining the best of two worlds: real time, label free measurements of live cells and flow cytometry. Thus, we measured the fluorescence of NADH as well as FAD of surface-stained cells under “Seahorse-like” conditions by flow cytometry on single cells. There are several other single-cell metabolic analyses based on flow cytometry such as SCENITH and MetFlow [25,52]. SCENITH measures the origin and abundance of ATP by proportional Puromycin incorporation, followed by antibody detection. MetFlow measures the abundance of rate-limiting metabolic enzymes by fluorescent antibody detection. In our experience, the technical highlights of NADH and FAD fluorescence measurements by flow cytometry are (i) the direct readout in live cells, enabling to detect heterogeneity within a cell population and relate it to certain phenotypes, (ii) the ease of use, (iii) economical aspects, (iv) the very small deviation of single data points and (v) the very high reproducibility.

With Jurkat cells, we reveal that changes in ORR, but also FAD and NAD(P)H fluorescence measured by flow cytometry correlate with OCR in a simulated MST. This is in line with a previous report correlating the ORR measured by 2-Photon microscopy with normalized OCR in breast cancer cells [53]. While extracellular flux analysis provides absolute numbers of pO_2_ and pH changes of a cell population over time, NAD(P)H and FAD fluorescence do not and consequently, these are relative measures, albeit on a single cell level. However, their ratio – ORR – is a normalized parameter, which provides direct information on the redox state of the cell independent of cell size and granularity, as previously shown (reviewed in [1]).

Aiming at correlating ORR and OCR, previous [20,21] and our own studies have shown that reduced respiratory activity correlates with reduced ORR ([53] and Jurkat cells in Figure 1E). In our hands, the ORR dropped after ATP synthase blockade also in non-mutant B cells. In contrast, B cells in which OxPhos is diminished by TwinkleK320E (DNT)-expression [6] (Figure 4) show a higher ORR. How can these data be reconciled? First, acute glucose availability is decisive. Acute glucose availability leads to a drop of the ORR in DNT B cells, as expected. Second, with regard to ATP Synthase inhibition, the composition of the ETC plays a role. MtDNA-encoded respiratory chain subunits, but not the nucleus-encoded ones, are less abundant in DNT B cells [6]. Notably, SDH is fully nucleus-encoded. In DNT-expressing B cells, fumarate is elevated [6], as is the case in human B cells with a gain-of-function mutation of SDHA [54]. Interestingly, SDH is fully active upon reduction of the respiratory chain and in the presence of ATP [14]. Consequently, when ATP is still present, as in activated DNT-expressing B cells which compensate by glycolysis [6], a decreased electron flow from succinate to oxygen would be compatible with a high reduction of the Ubiquinone pool based on a relative increase in SDH-activity. This could explain the increase in FAD and ORR in DNT B cells. What's more, cells with defective mtDNA may build up a high FAD content through compensatory upregulation of alternative metabolic pathways. Therefore, the interpretation of ORR with respect to mitochondrial function has to be performed with caution: Both a reduction and an increase of the ORR can point to dysfunction of OxPhos complexes, depending on the medium and on the relative activity of ETC complexes I–V. In that regard it is interesting to note that LPS activated GLUT1KO B cells show a higher ORR, confirming mitochondrial malfunction [37]. Independent of these considerations, it is clear that provoked metabolic defects of glycolysis and OxPhos in mouse B cells are mirrored by altered ORR.

This notion encouraged us to test reciprocally whether putative metabolic alterations can be uncovered in an unbiased manner by our approach. Therefore, based on the idea that RA lymphocytes are metabolically different [45,[55], [56], [57], [58]], we assessed B and T lymphocytes in PBMC from healthy subjects and RA patients. In accordance with published data, we found reduced non-switched memory B cells in RA patients [59]. Assessing beforehand different lymphocyte populations from HD, the ORR was lowest in switched memory B cells over the whole assay, followed by non-switched and naïve B cells, and finally CD4^+^ T cells. Of note, FCCP treatment reduced glucose-induced FAD much less in naïve and non-switched vs. switched memory B cells. These data suggest that resting human switched memory B cells in the very same environment as naïve B cells are metabolically more active based on coupled glucose-FAD metabolism, thereby possibly being poised to facilitate activation and survival [60]. These data are compatible with the finding that human memory B cell differentiation into plasma cells requires glucose based metabolic reprogramming [41]. Hence, kickstarted glycolytic capacity in memory B cells may facilitate plasma cell differentiation.

Unexpectedly, RA lymphocytes (naïve B cells, CD4^+^ T cells, non-switched and switched memory B cells) displayed a dampened increase of FAD upon acute Glucose availability than the corresponding HD lymphocytes; naïve B cells and T cells from RA patients also showed less NADH. CD4^+^ T cells, naive and switched memory B cells from RA patients revealed a less efficient rise of FAD and NADH upon combined Glucose and FCCP treatment, suggesting there is less coupling of glucose to respiration in these cells from RA donors. These data coincide with reports showing that B cells from ACPA-positive RA patients differentiate less well into plasma cells [56,57] but they stand in contrast to findings that B cells from RA patients show a more activated phenotype based on CD86 and CD69 expression [45,[55], [56], [57]]. More work is needed to reconcile those findings. A limitation of our work as it stands is the limited sample size and the possibility that anti-inflammatory therapy RA patients have been exposed to, such as Prednisolone, may exert metabolic effects. For instance, glucocorticoids re-establish the respiratory chain and Krebs cycle activity in otherwise pro-inflammatory macrophages [61]. In fact, the ORR tended to be higher in resting lymphocytes of RA patients (except of non-switched memory B cells), which could be explained by a more oxidative metabolism elicited via medication. However, only 2 out of the assessed RA patients received glucocorticoids and treatments were heterogeneous. Thus, the differences we observed are likely not due to glucocorticoid treatment. Notwithstanding, it will be mandatory to address the influence of anti-inflammatory treatments, such as glucocorticoids, on the ORR in lymphocytes isolated from healthy or acutely treated donors. This might enable a rapid read-out to predict the efficacy of medication. Future experiments should include pre RA patients devoid of treatment. In addition to glucose, the effects of fatty acids, amino acids (albeit abundant in the culture medium) or ketone bodies as valuable sources of energy for lymphocytes might be relevant [40,62,63]. Accordingly, changes in the ORR of murine B cells and Jurkat cells in response to acute Glutamine and Palmitate availability were depicted by us by flow cytometry. Lastly, more specialized populations associated with different autoimmune disorders, such as ABC or DN B cells [[64], [65], [66], [67]], could be analyzed in the future. On top, transformed B cells represent different entities awaiting further metabolic characterization. For instance, diffuse large B cell lymphoma (DLBCL) tumors are heterogeneous [68]. Furthermore, whereas mutated chronic lymphocytic leukemia (CLL) and indolent follicular lymphoma cells show a predisposition to fatty acid oxidation and Oxphos [69,70], the metabolism of CLL cells can be modulated by stromal cells in co-culture [71]. In line, DLBCL, Reed-Sternberg cells and multiple myeloma cells undergo many mutual metabolic interactions with surrounding stroma cells, macrophages and others [72]. Thus, depicting the ORR of different cell types from mixed lymphoma-stroma cultures and fresh explants by flow cytometry might shed light on metabolic mechanisms driving lymphoma growth. In this regard, spectral flow cytometry might become very helpful.

In summary, we have developed a method that couples surface marker-based cell identification by flow cytometry with label-free real time metabolic analyses. This technique sets the stage for the future application of sophisticated combinations of surface markers to track down the metabolism in particular cell lineages in different diseases.

## CRediT authorship contribution statement

**Ariful Haque Abir:** Writing – review & editing, Writing – original draft, Visualization, Validation, Methodology, Investigation, Formal analysis, Data curation, Conceptualization. **Leonie Weckwerth:** Writing – review & editing, Data curation. **Artur Wilhelm:** Writing – review & editing, Data curation. **Jana Thomas:** Writing – review & editing. **Clara M. Reichardt:** Writing – review & editing, Formal analysis, Data curation. **Luis Munoz:** Writing – review & editing. **Simon Völkl:** Writing – review & editing, Validation. **Uwe Appelt:** Writing – review & editing, Validation. **Markus Mroz:** Writing – review & editing. **Raluca Niesner:** Writing – review & editing. **Anja Hauser:** Writing – review & editing. **Rebecca Sophie Fischer:** Writing – review & editing. **Katharina Pracht:** Writing – review & editing. **Hans-Martin Jäck:** Writing – review & editing. **Georg Schett:** Writing – review & editing, Project administration. **Gerhard Krönke:** Writing – review & editing. **Dirk Mielenz:** Writing – review & editing, Writing – original draft, Validation, Supervision, Resources, Project administration, Investigation, Funding acquisition, Formal analysis, Conceptualization.

## Declaration of competing interest

The authors declare that they have no known competing financial interests or personal relationships that could have appeared to influence the work reported in this paper.

## Data Availability

Data will be made available on request.
