# Supplementary material for: Metabolic profiling of single cells by exploiting NADH and FAD fluorescence via flow cytometry
Source: Mol Metab. 2024 Jul 4;87:101981. doi: 10.1016/j.molmet.2024.101981 (PMC11300934; doi:10.1016/j.molmet.2024.101981)
Supplement: Multimedia component 1 [file mmc1.pdf]

Abir et al. – Supplemental material

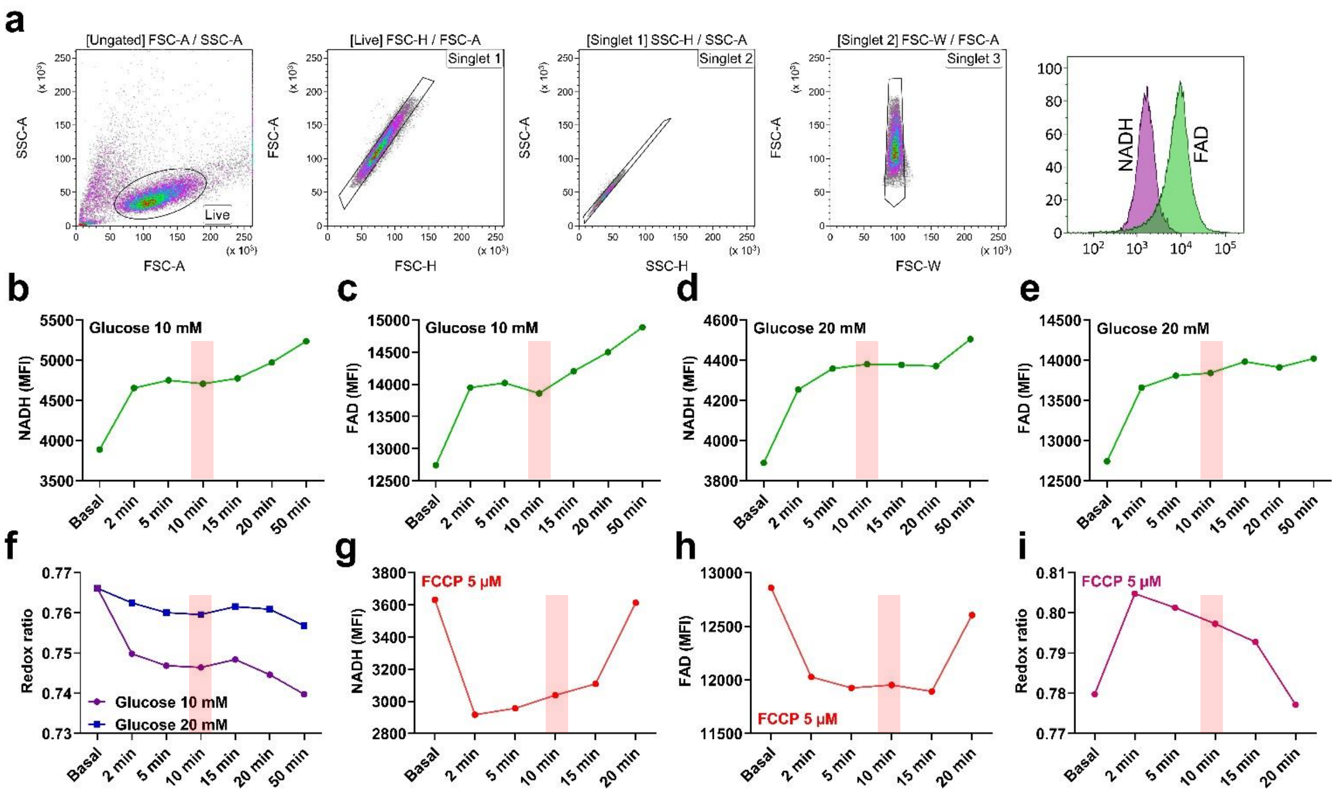

Figure S1. Gating strategy and establishment of basic parameters for fluorescence detection of Jurkat T cells

Full gating strategy for the analysis of NADH and FAD fluorescence detection of Jurkat T cells. b-e. Jurkat T cells were placed in glucose-free medium. Glucose was added and NADH as well as FAD fluorescence were measured by flow cytometry. Time course and titration of glucose addition and its effects on NADH and FAD fluorescence in Jurkat T cells are depicted. f. Kinetics of the optical redox ratio in response to different glucose concentrations in Jurkat T cells. g, h. Jurkat T cells were placed in glucose-free medium. Carbonyl cyanide m-chlorophenyl hydrazine (FCCP) was added and NADH as well as FAD fluorescence were measured by flow cytometry. Time course and titration of glucose addition and its effects on NADH and FAD fluorescence in Jurkat T cells are depicted. i. Kinetics of the optical redox ratio in response to FCCP in Jurkat T cells.

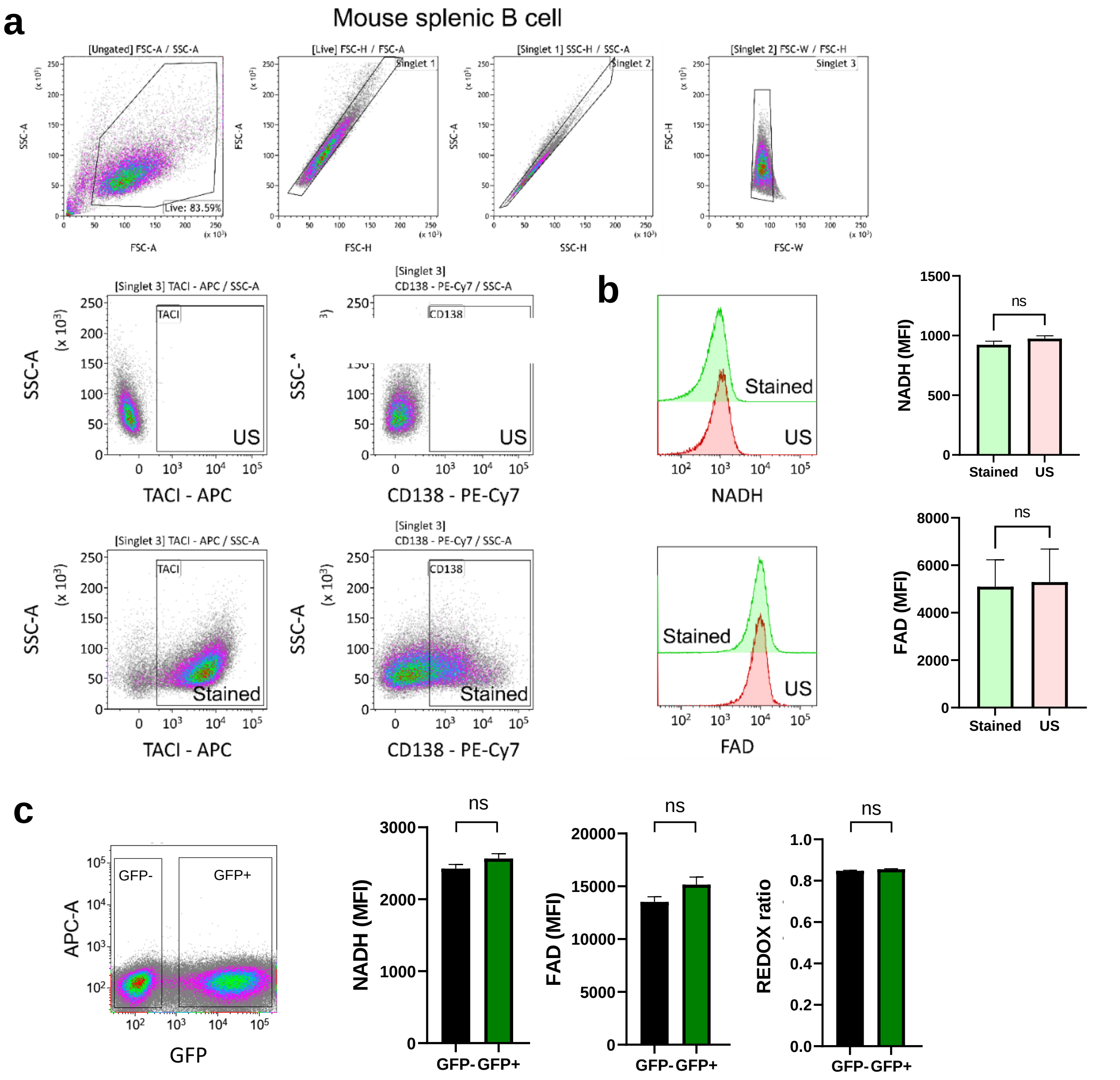

Figure S2. Gating strategy for mouse B cells

a. B cells from wildtype mice (C57Bl/6) were stimulated for 3 days with lipopolysaccharide, surface-stained as indicated and analyzed by flow cytometry. b. NADH and FAD fluorescence of anti CD138, TACI as well as GL7-stained and unstained (US) activated B cells (singlet gate from b). Representative histograms and mean -/+ SD of 5 independent experiments, Wilcoxon t-test. c. B cells from wildtype mice (C57Bl/6) were stimulated for 1 day with lipopolysaccharide. Activated B cells were infected with a GFP-encoding retrovirus and analyzed 24h later for GFP expression, NADH and FAD fluorescence. Data are presented as mean -/+ SD of three B cell cultures from three different mice, paired t-test.

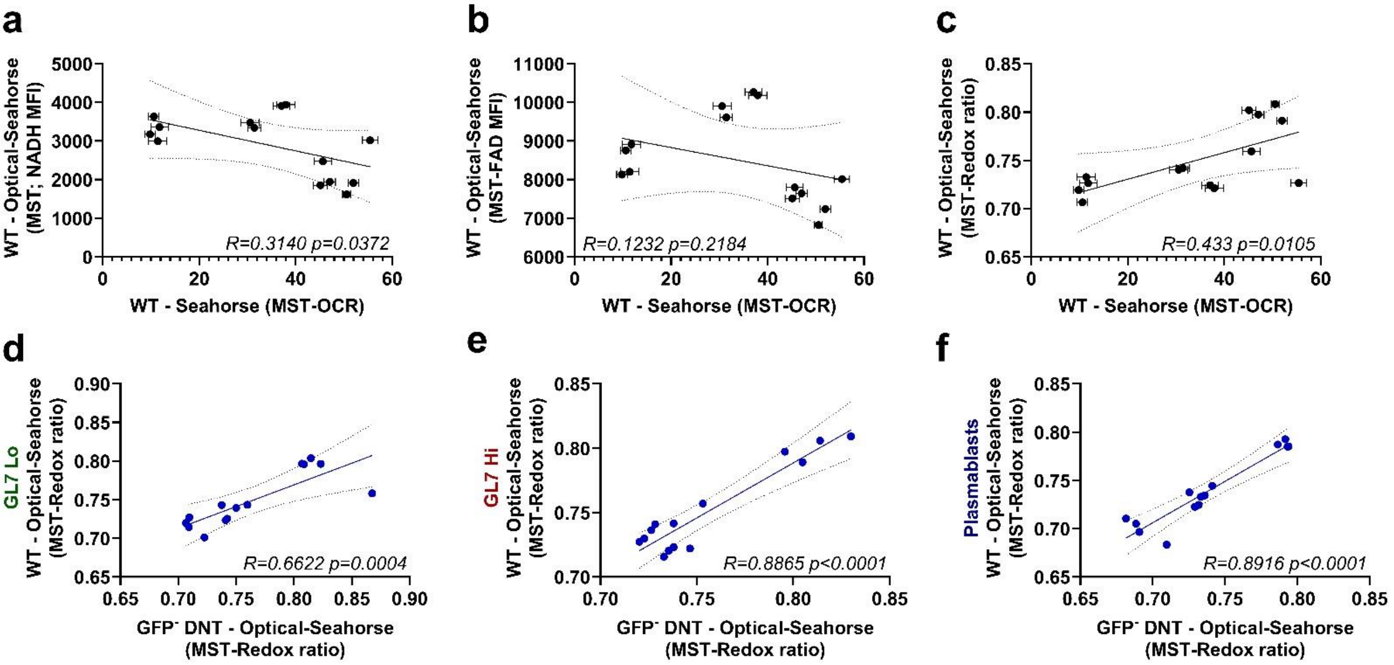

Figure S3. Correlation analyses of Mito Stress Test oxygen consumption rates and optical redox ratios

B cells from wildtype mice (C57Bl/6) were stimulated for 3 days with lipopolysaccharide, surface-stained as indicated and analyzed by flow cytometry or Seahorse Mito Stress Test (MST).

a. The linear regression analysis shows that the MST-NADH measurement of WT murine cells are not directly correlated to OCR of Seahorse MST data of the same WT murine cells with a R-squared value of 0.3140 (p=0.0.0372, n=2).

b. The linear regression analysis shows that the MST-FAD measurement of WT murine cells are not directly correlated to OCR of Seahorse MST data of the same WT murine cells with a R-squared value of 0.1232,(p=0.0.2184, n=2).

c. The linear regression analysis shows that the MST-ORR measurement of WT murine cells are not directly correlated to OCR of Seahorse MST data of the same WT murine cells with a R-squared value of 0.4330 (p=0.0.0105, n=2).

d. The linear regression analysis shows that the MST-ORR measurement of WT $GL7^{Lo}$ murine cells are directly correlated to the GFP^- DNT murine $GL7^{Lo}$ cells with a R-squared value of 0.6622 (p=0.0004, n=2).

e. The linear regression analysis shows that the MST-ORR measurement of WT $GL7^{Hi}$ murine cells are directly correlated to the GFP^- DNT murine $GL7^{Hi}$ cells with a R-squared value of 0.8865 (p<0.0001, n=2).

e. The linear regression analysis shows that the MST-ORR measurement of WT murine plasmablasts are directly correlated to the GFP^- DNT murine plasmablast with a R-squared value of 0.8916 (p<0.0001, n=2).

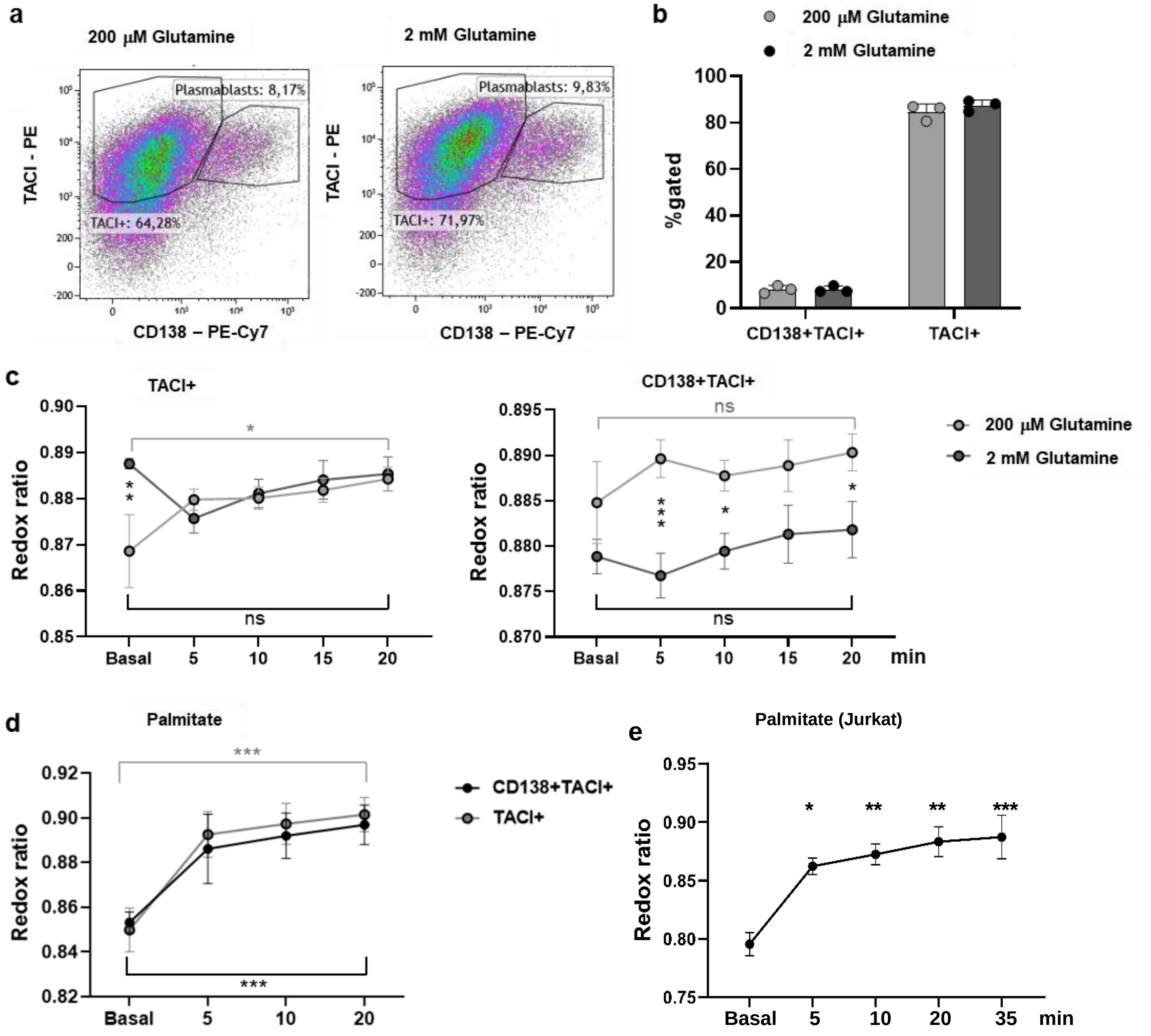

Figure S4. Effect of Glutamine and Palmitate on the optical redox ratio of LPS activated B cells
B cells from wildtype mice (C57Bl/6) were stimulated for 3 days with lipopolysaccharide with reduced (200 µM) or standard concentrations (2 mM) of Glutamine in full medium and surface-stained as indicated, a. Representative flow cytometry dot blots. b. frequency of CD138+TACI+ and TACI+ cells (each dot represents one mouse), mean -/+ SD. c. Cells were stimulated with 2 mM Glutamine and analyzed by flow cytometry. The optical redox ratio is depicted. d. Cells cultured in 2 mM Glutamine were stimulated with Palmitate and analyzed by flow cytometry. The optical redox ratio is depicted. Data are presented as mean -/+ SD of three B cell cultures from three different mice, 2-way ANOVA with Tukey's multiple comparisons test. * $p < 0.05$, ** $p < 0.02$, *** $p<0.001$, ns, not significant, basal vs. 20 min. Vertical *: difference between 200 µM Glutamine and 2 mM Glutamine. e. Jurkat were treated with 200µM Palmitate+BSA conjugate followed by an hour of starvation. Afterwards, the NADH and FAD fluorescence was measured with the flow cytometry at the indicated timepoint. The ORR was calculated and analyzed with One-way ANOVA-RM, *p=0.01, **p=0.001-0.003, ***P < 0.001 (Turkey's multiple comparison test, basal vs. 5-35 min), n=4, graph is presented as mean ±SEM.

a

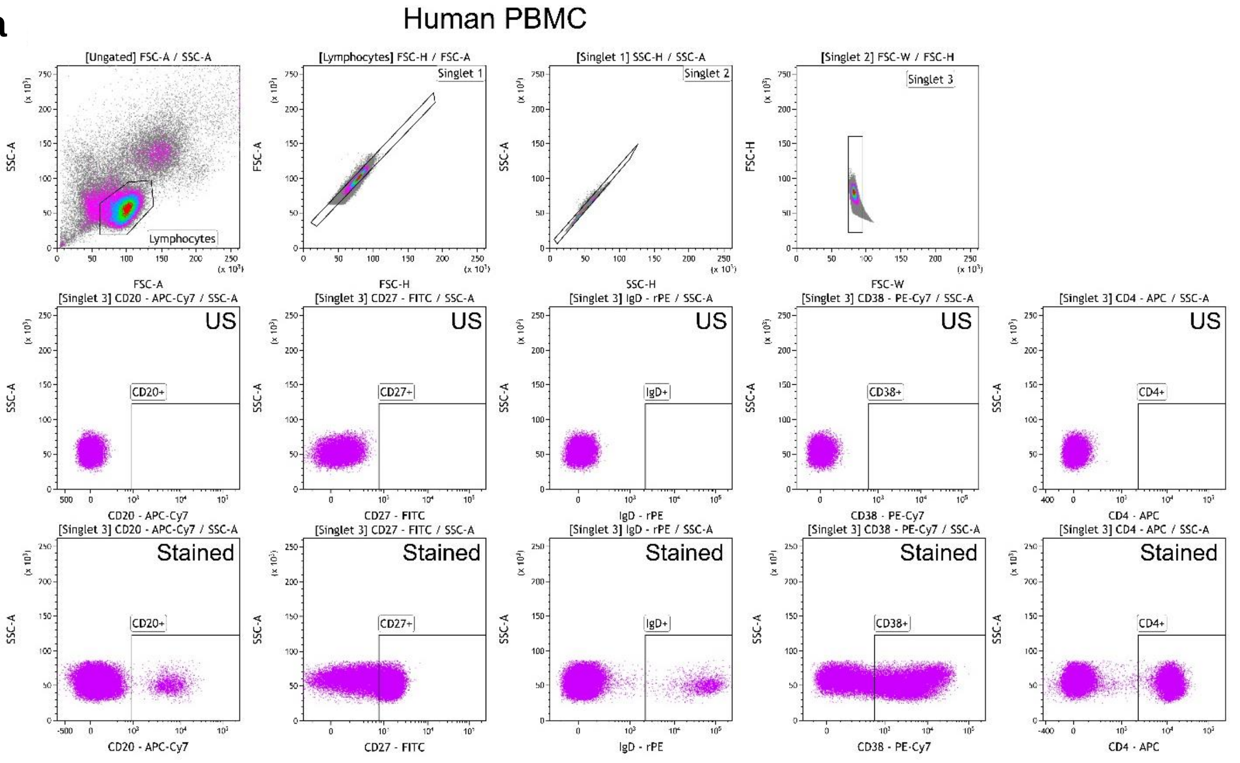

b

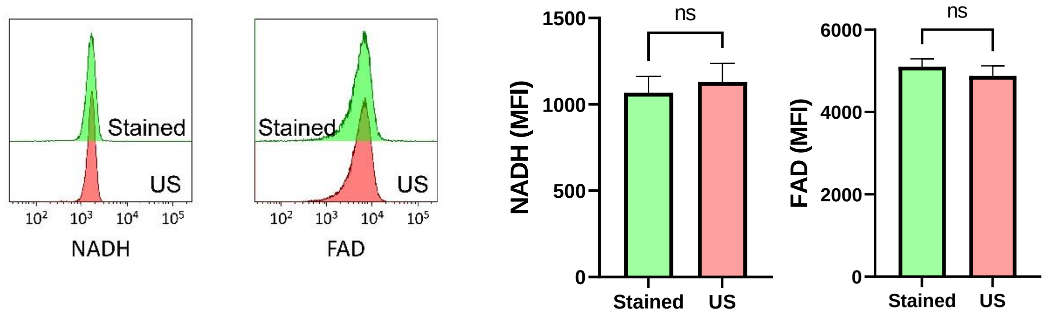

Figure S5. Gating strategy for human peripheral blood mononuclear cells

a. Peripheral blood mononuclear cells (PBMC) from healthy donors were surface-stained as indicated and analyzed by flow cytometry. b. NADH and FAD fluorescence of anti CD20, CD27, IgD and CD4-stained and unstained (US) PBMC (singlet gate from a). Representative data and mean -/+ SD of 9 different donors, Wilcoxon t-test.

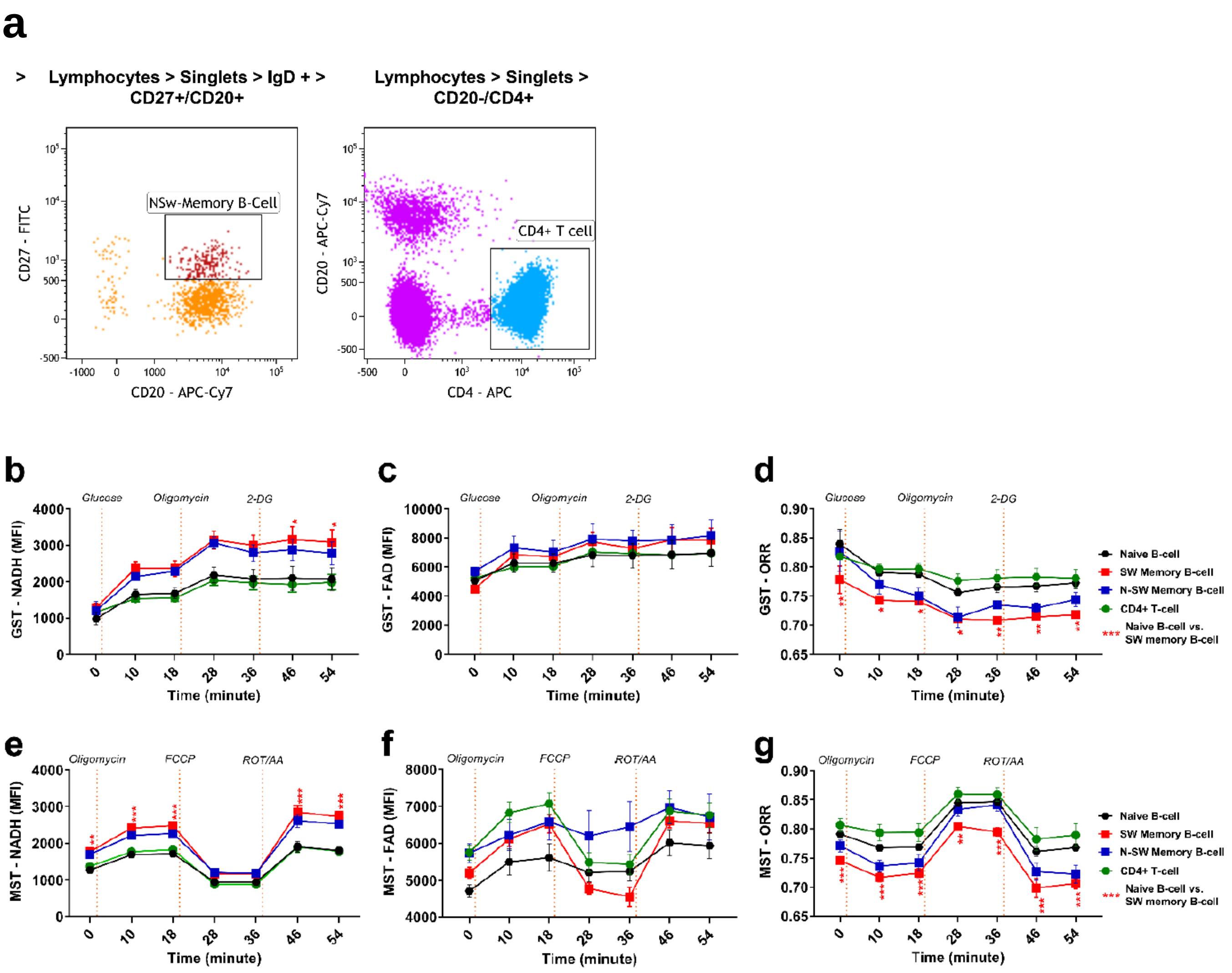

Figure S6. Simultaneous determination of optical redox ratios in different populations of human peripheral blood mononuclear cells

Peripheral blood mononuclear cells (PBMC) from a healthy donor were surface-stained as indicated and analyzed by flow cytometry. In the representative dot plot, IgD^+,$CD27^+$,$CD20^+$ cells are Non-Switched (NSW) memory B-cells and CD4 indicates T helper cells.

b, c. PBMC from HD were surface-stained as indicated in (a) and placed in glucose-free medium. Glucose, oligomycin and 2-Deoxyglucose (2-DG) were added at indicated time points and NADH as well as FAD fluorescence was measured by flow cytometry. Two-way ANOVA repeated measures, *p=0.03-0.04 (Bonferroni multiple comparison test), n = 5, values are mean ± SEM.

d. The redox ratio [FAD]/([FAD]+[NAD(H)]) of PBMC from the experiment shown in b, c. Two-way ANOVA repeated measures, *p=0.0189-0.0315, **p=0.0012-0.0075 (Bonferroni multiple comparison test), n = 5, values are mean ± SEM.

e, f. PBMC from HD were surface-stained as indicated in (a) and placed in Mito Stress Test medium. Oligomycin, carbonyl cyanide m-chlorophenyl hydrazine (FCCP), Rotenone/Antimycin were added at indicated time points and NADH and FAD fluorescence were measured by flow cytometry. Two-way ANOVA repeated measures, **p=0.003, ***p ≤ 0.001 (Bonferroni multiple comparison test), n = 5, values are mean ± SEM.

g. The redox ratio [FAD]/([FAD]+[NAD(H)]) of PBMC from the experiment shown in e, f. Two-way ANOVA repeated measures, **p=0.002, ***p ≤ 0.001 (Bonferroni multiple comparison test), n = 5, values are mean ± SEM.

Sex	Age	CCP peptide (RE/mL)	RF (IE/mL)	Treatment
F	54	138	323	Methotrexate, Roactemra
F	40	88	42	Prednisolone, Certolizumab
F	61	50	170	Leflunomide, Hydroxychloroquine
F	58	no data	no data	Mycophenolate, Prednisolone
F	43	65	6	Humira, Methotrexate, Folate
F	76	367	51	Methotrexate
F	79	271	220	Methotrexate
F	77	128	44	Methotrexate, Folate
F	54	138	323	Methotrexate, Roactemra

Supplementary Table 1: Patient information
Blood was drawn during regular visits and blood parameters were determined. The table depicts sex (F: female), age (years), the relative units (RE) and international units (IE) of anti citrullinated peptide antibodies and rheumatoid factor, as well as treatment. Roactemra (Tocilizumab, anti IL-6 antibody), Certolizumab (Fab fragment, anti TNF-α), Humira (Adalimumab, anti TNF-α).
